# Supplementary material for: Mitochondria from osteolineage cells regulate myeloid cell-mediated bone resorption
Source: Nat Commun. 2024 Jun 14;15:5094. doi: 10.1038/s41467-024-49159-3 (PMC11178781; doi:10.1038/s41467-024-49159-3)
Supplement: Supplementary file 1 — Supplementary Information [file 41467_2024_49159_MOESM1_ESM.pdf]

Mitochondria from osteolineage cells regulate myeloid cell-mediated bone resorption

Peng Ding[1,2†], Chuan Gao[1,2†], Jian Zhou[1,2†], Jialun Mei[1,2†], Gan Li[1,2], Delin Liu[1,2], Hao Li[1,2], Peng Liao[1,2], Meng Yao[1,2], Bingqi Wang[1,2], Yafei Lu[1,2], Xiaoyuan Peng[1], Chenyi Jiang[1], Jimin Yin[1], Yigang Huang[1], Minghao Zheng[3], Youshui Gao[1]*, Changqing Zhang[1,2]*, Junjie Gao[1,2]*

[1]Department of Orthopaedics, Shanghai Sixth People's Hospital Affiliated to Shanghai Jiao Tong University School of Medicine, Shanghai, 200233, China.
[2]Institute of Microsurgery on Extremities, Shanghai Sixth People's Hospital Affiliated to Shanghai Jiao Tong University School of Medicine, Shanghai 200233, China.
[3]Centre for Orthopaedic Translational Research, Medical School, University of Western Australia, Nedlands, Western Australia 6009, Australia.

Corresponding Author

*To whom correspondence should be addressed:
Junjie Gao: colingjj@163.com
Changqing Zhang: zhangcq@sjtu.edu.cn
Youshui Gao: gaoyoushui@sjtu.edu.cn

[†] These authors contributed equally, *Correspondence

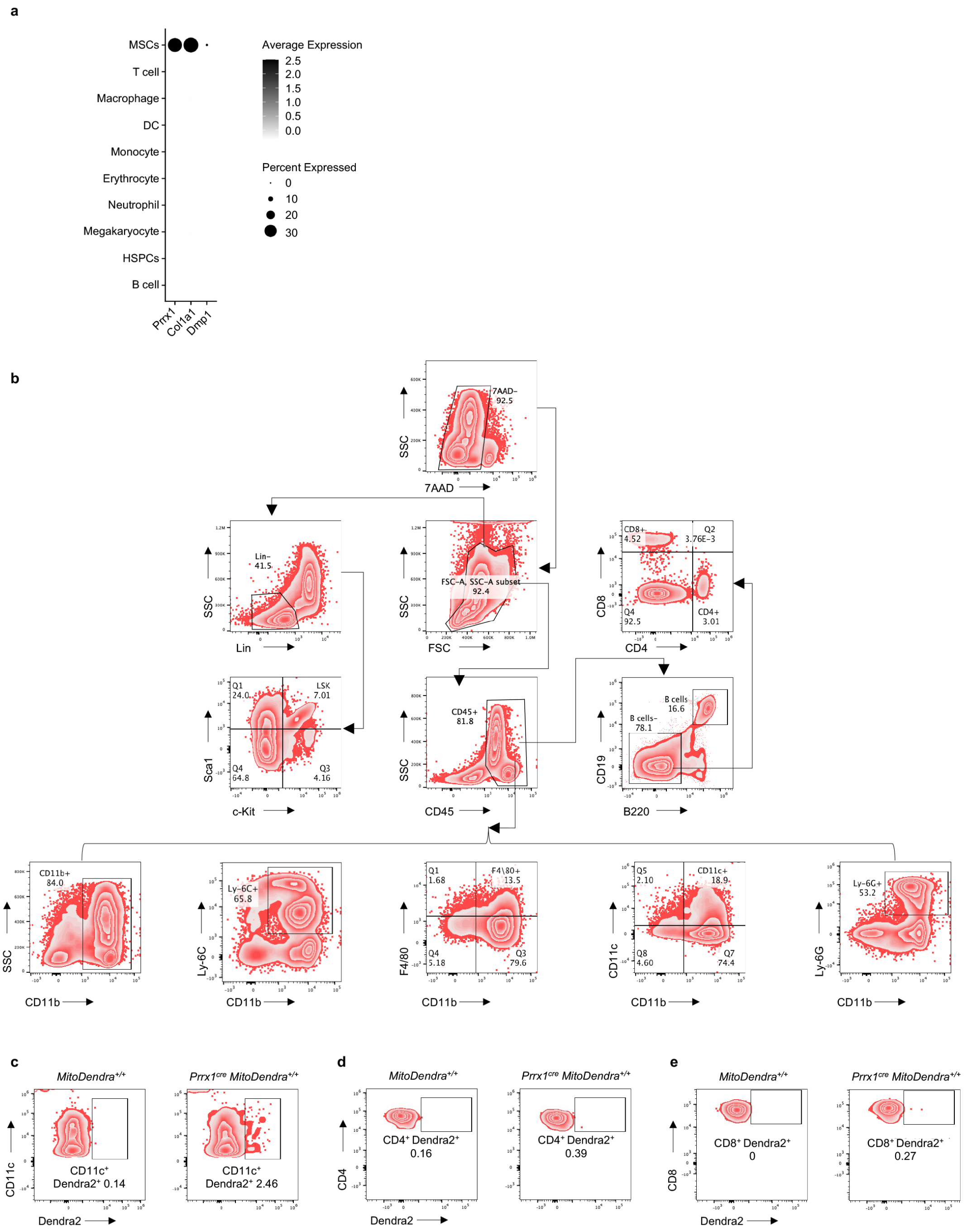

Supplementary Fig. 1 | Osteolineage transfer mitochondria to myeloid cells. a, Sc-RNA sequencing showing no obvious expression of *Prrx1*, *Col1a1* or *Dmp1* in other clusters of bone marrow cells except the MSC cluster. **b,** Representative images of flow cytometry gating of HSPCs (Lin$^-$Sca1$^+$c-Kit$^+$), CD4$^+$ T cells (CD45$^+$B220$^-$CD19$^-$CD4$^+$CD8$^-$), CD8$^+$ T cells (CD45$^+$B220$^-$CD19$^-$CD4$^-$CD8$^+$), B cells (CD45$^+$B220$^+$CD19$^+$), CD11b$^+$ myeloid cells (CD45$^+$CD11b$^+$), monocytes (CD45$^+$CD11b$^+$Ly-6C$^+$), macrophages (CD45$^+$CD11b$^+$F4/80$^+$), DCs (CD45$^+$CD11b$^+$CD11c$^+$) and neutrophils (CD45$^+$CD11b$^+$Ly-6G$^+$). **c,** Representative images of flow cytometry showing mitochondria transfer to DCs in *Prrx1^{cre} MitoDendra$^{+/+}$* mice (n = 6 per group). **d, e,** Representative images of flow cytometry showing no mitochondria transfer to CD4$^+$ **(d)** and CD8$^+$ **(e)** T cells in *Prrx1^{cre}*

MitoDendra$^{+/+}$ mice (n = 6 per group).

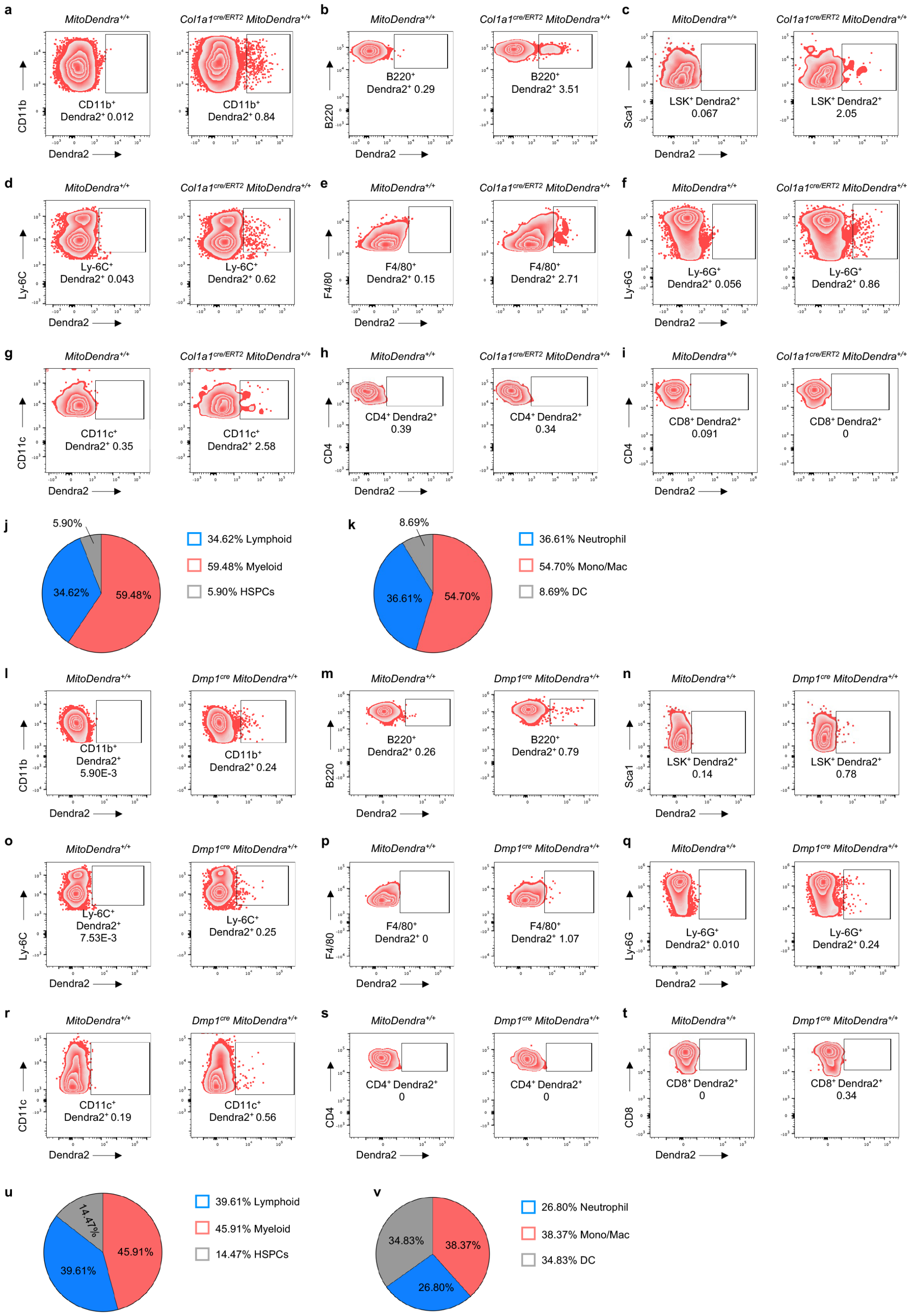

Supplementary Fig. 2 | Osteolineage cells transfer mitochondria to myeloid cells. **a-c,** Representative images of flow cytometry showing mitochondria transfer to CD11b^{+} myeloid cells **(a)**, B220^{+} lymphoid cells **(b)** and HSPCs **(c)** in *Col1a1$^{cre/ERT2}$ MitoDendra$^{+/+}$* mice (*n* = 6 per group). **d, e,** Representative images of flow cytometry showing mitochondria transfer to monocytes **(d)** and macrophages **(e)** in *Col1a1$^{cre/ERT2}$ MitoDendra$^{+/+}$* mice (*n* = 6 per group). **f, g,** Representative images of flow cytometry

showing mitochondria transfer to neutrophils **(f)** and DCs **(g)** in *Col1a1*$^{cre/ERT2}$ *MitoDendra*$^{+/+}$ mice (n = 6 per group). **h, i,** Representative images of flow cytometry showing no mitochondria transfer to CD4^{+} **(h)** and CD8^{+} **(i)** T cells in *Col1a1*$^{cre/ERT2}$ *MitoDendra*$^{+/+}$ mice (n = 6 per group). **j,** The proportion of cell types that received osteolineage mitochondria in *Col1a1*$^{cre/ERT2}$ *MitoDendra*$^{+/+}$ mice (n = 4 per group). **k,** The proportion of subcluster of myeloid cells that received osteolineage mitochondria in *Col1a1*$^{cre/ERT2}$ *MitoDendra*$^{+/+}$ mice (n = 6 per group). **l-n,** Representative images of flow cytometry showing mitochondria transfer to CD11b^{+} myeloid cells **(l)**, B220^{+} lymphoid cells **(m)** and HSPCs **(n)** in *Dmp1*^{cre} *MitoDendra*$^{+/+}$ mice (n = 5 per group). **o, p,** Representative images of flow cytometry showing mitochondria transfer to monocytes **(o)** and macrophages **(p)** in *Dmp1*^{cre} *MitoDendra*$^{+/+}$ mice (n = 5 per group). **q, r,** Representative images of flow cytometry showing mitochondria transfer to neutrophils **(q)** and DCs **(r)** in *Dmp1*^{cre} *MitoDendra*$^{+/+}$ mice (n = 5 per group). **s, t,** Representative images of flow cytometry showing no mitochondria transfer to CD4^{+} **(s)** and CD8^{+} **(t)** T cells in *Dmp1*^{cre} *MitoDendra*$^{+/+}$ mice (n = 5 per group). **u,** The proportion of cell types that received osteolineage mitochondria in *Dmp1*^{cre} *MitoDendra*$^{+/+}$ mice (n = 5 per group). **v,** The proportion of subclusters of myeloid cells that received osteolineage mitochondria in *Dmp1*^{cre} *MitoDendra*$^{+/+}$ mice (n = 5 per group). Source data are provided as a Source Data file.

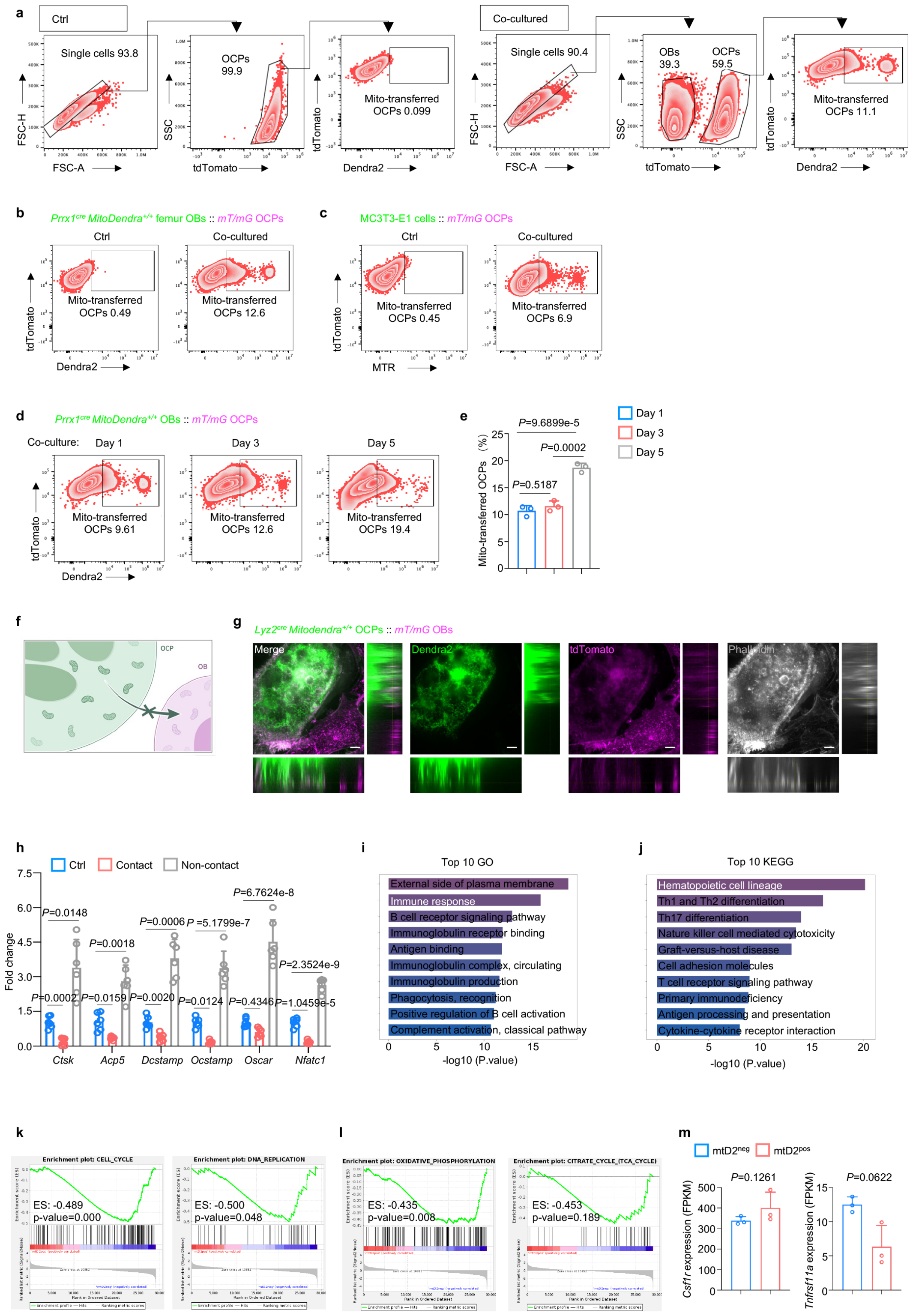

Supplementary Fig. 3 | Osteolineage cells transfer mitochondria to osteoclastic lineage cells and inhibit osteoclast activity. a, Representative images of flow cytometry gating mitochondria transferred OCPs. **b,** Representative images of flow cytometry demonstrating the transfer of Dendra2-positive mitochondria from *Prrx1*^{cre} *MitoDendra*$^{+/+}$ femoral OBs to *mT/mG* OCPs. **c,** Representative images of flow cytometry demonstrating the transfer of MitoTracker labeled mitochondria from

MC3T3-E1 cells to *mT/mG* OCPs. **d, e,** Representative images of flow cytometry **(d)** and analysis **(e)** showing the increased transfer of Dendra2-positive mitochondria from OBs to OCPs with the elongation of coculture time from Day 1, Day 3 to Day 5 (n = 3 per group). **f,** Schematic of no mitochondria transfer from OCPs to OBs. This figure was created by P.D. and cartoonized by Mr. Zihao Li. **g,** Representative images showing no polarization of mitochondria distribution in OCPs and transferred mitochondria in OBs. Scale bar, 25 μm. **h,** The relative expression of osteoclastic signature genes with or without OB direct or indirect coculture under RANKL stimulation (n = 6 per group). **i,** The top 20 GO pathways showing that immune-related processes were enriched in myeloid cells after mitochondria transfer from osteolineage cells in vivo. **j,** The top 20 KEGG analysis showing the enrichment of genes associated with hematopoietic cell lineage pathway after mitochondria transfer from osteolineage cells in vivo. **k,** GSEA showing the enrichment of genes associated with the cell cycle and DNA replication. **l,** GSEA showing the enrichment of genes associated with oxidative phosphorylation and the citrate cycle. **m,** The relative expression of *Csf1r* and *Tnfrsf11a* between mtD2pos and mtD2neg OCPs derived from RNA sequencing. Data are presented as the mean ± s.d., with biologically individual data points shown. *P* values were determined by ordinary one-way ANOVA test with Tukey's multiple comparisons (*Ocstamp*, *Oscar* and *Nfatc1* of **h**), Brown-Forsythe and Welch ANOVA tests with Dunnett's T3 multiple comparisons (*Ctsk, Acp5* and *Dcstamp of* **h**), unpaired two-tailed Student's *t*-test with Welch's correction (*Tnfrsf11a* of **m**), unpaired two-tailed Student's *t*-test **(m)**. Source data are provided as a Source Data file.

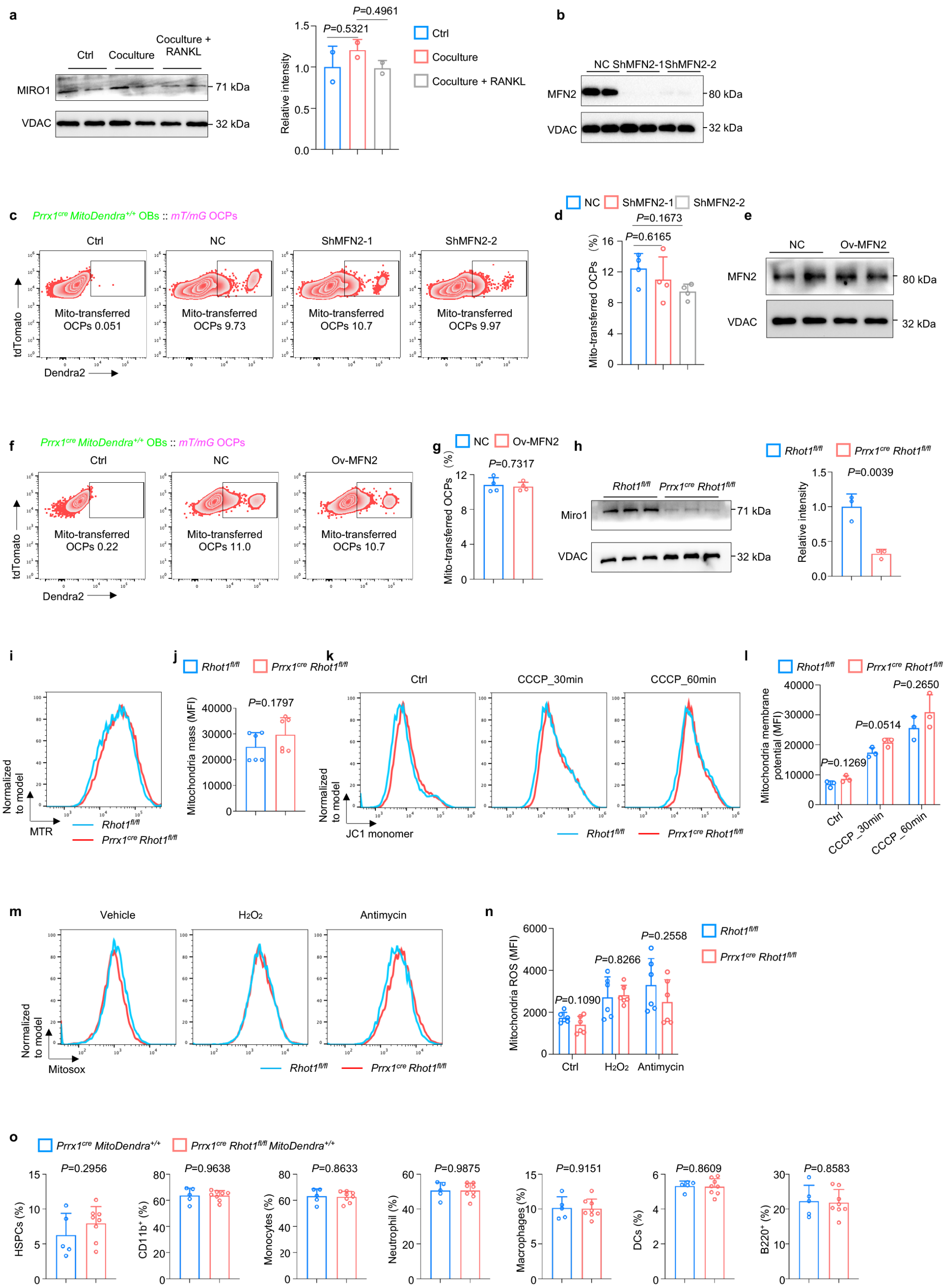

Supplementary Fig. 4 | MIRO1 mediates the mitochondria transfer from osteolineage to osteoclastic lineage cells. a, Western blot and quantification of MIRO1 expression in OBs with or without direct coculture and indirect coculture with OCPs under RANKL stimulation (n = 2 per group). **b,** Western blot of OBs transduced with *Mfn2* shRNA lentiviral particles. **c, d,** Representative images of flow cytometry **(c)** and analysis **(d)** showing no change in the transfer of Dendra2-positive mitochondria from OBs to OCPs after MFN2 downregulation (n = 4 per group). **e,** Western blot of OBs

transduced with *Mfn2* shRNA lentiviral particles. **f, g,** Representative images of flow cytometry **(f)** and analysis **(g)** showing no change in the transfer of Dendra2-positive mitochondria from OBs to OCPs after MFN2 upregulation (*n* = 4 per group). **h,** Western blot and quantification demonstrating the knockout of MIRO1 in osteoblast in vitro in *Prrx1*^{cre} *Rhot1*$^{fl/fl}$ mice (*n* = 3 per group). **i, j,** Representative images of flow cytometry **(i)** and analysis **(j)** showing no difference in OB mitochondria mass between *Rhot1*$^{fl/fl}$ and *Prrx1*^{cre} *Rhot1*$^{fl/fl}$ mice (*n* = 6 per group). **k, l,** Representative images of flow cytometry **(k)** and analysis **(l)** showing no difference in OB mitochondria membrane potential with CCCP stimulation between *Rhot1*$^{fl/fl}$ and *Prrx1*^{cre} *Rhot1*$^{fl/fl}$ mice (*n* = 3 per group). **m, n,** Representative images of flow cytometry **(m)** and analysis **(n)** showing no difference in OB mitochondria ROS with H_2O_2 and antimycin stimulation between *Rhot1*$^{fl/fl}$ and *Prrx1*^{cre} *Rhot1*$^{fl/fl}$ mice (*n* = 6 per group). **o,** Flow cytometry analysis showing that there was no significant difference in the number of HSPCs, myeloid cells and B220^{+} lymphoid cells between *Prrx1*^{cre} *MitoDendra*$^{+/+}$ and *Prrx1*^{cre} *Rhot1*$^{fl/fl}$ *MitoDendra*$^{+/+}$ mice (*n* = 5 in *Prrx1*^{cre} *MitoDendra*$^{+/+}$ group and *n* = 8 in *Prrx1*^{cre} *Rhot1*$^{fl/fl}$ *MitoDendra*$^{+/+}$ group). Data are presented as the mean ± s.d., with biologically individual data points shown. *P* values were determined by ordinary one-way ANOVA with Tukey's multiple comparisons test **(a, d)**, unpaired two-tailed Student's *t*-test **(g, h, l, n, o)**, and Mann-Whitney U test **(j)**. Source data are provided as a Source Data file.

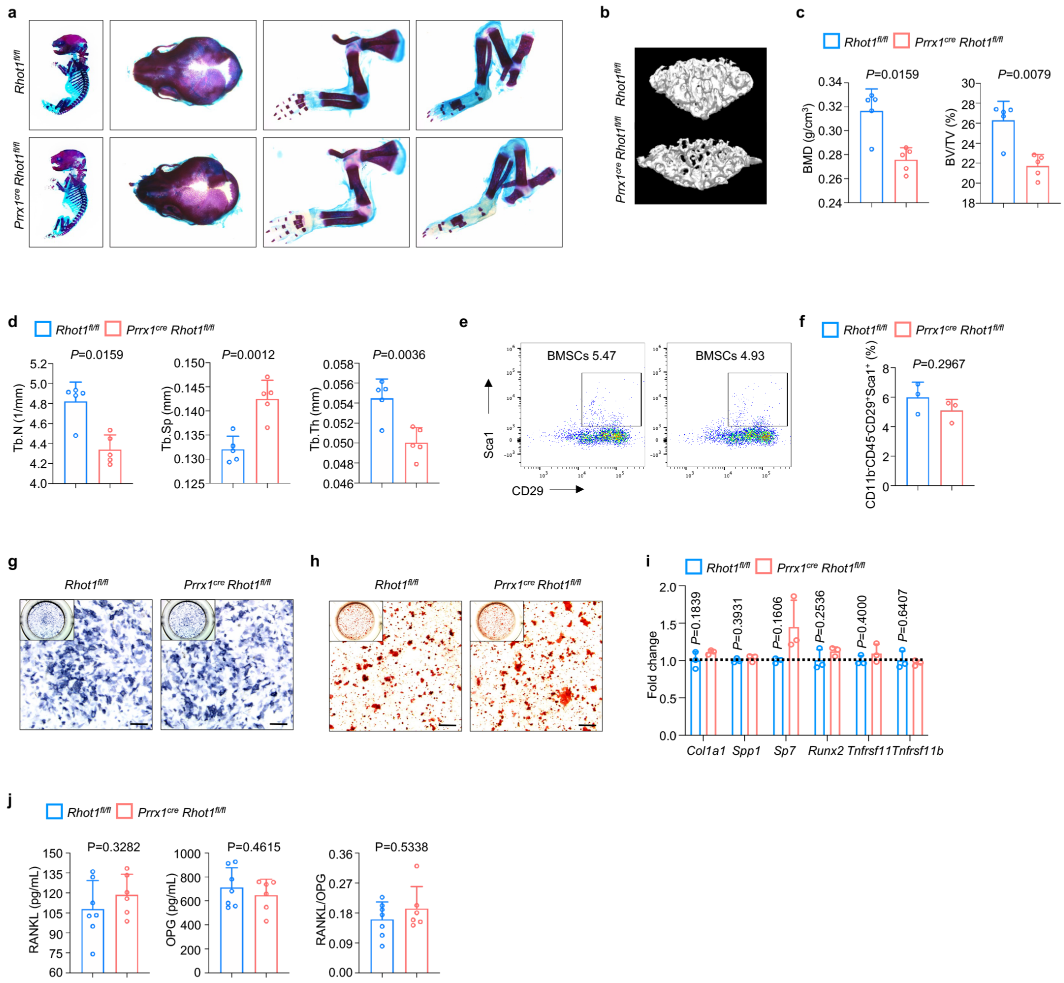

Supplementary Fig. 5 | Impaired mitochondria transfer from osteolineage to osteoclastic lineage cells results in osteoclastic bone loss. a, Whole mount skeletal staining of $Rhot1^{fl/fl}$ and $Prrx1^{cre}$ $Rhot1^{fl/fl}$ mice at P0 by Alizarin red/Alcian blue staining. **b-d,** Representative µCT reconstructed images of male $Rhot1^{fl/fl}$ and $Prrx1^{cre}$ $Rhot1^{fl/fl}$ mouse spines at 8 weeks **(b)** and trabecular microstructural parameters (BMD, BV/TV, Tb.N, Tb.Sp and Tb.Th) **(c, d)** derived from µCT analysis (n = 5 per group). **e, f,** Representative images of flow cytometry **(e)** and analysis **(f)** showing no difference in the number of osteoprogenitors ($CD11b^{-}CD45^{-}CD29^{+}Sca1^{+}$) in $Rhot1^{fl/fl}$ and $Prrx1^{cre}$ $Rhot1^{fl/fl}$ mice (n = 3 per group). **g, h,** ALP **(g)** and ARS **(h)** staining of osteogenesis in vitro of sorted BMSCs from $Rhot1^{fl/fl}$ and $Prrx1^{cre}$ $Rhot1^{fl/fl}$ mice, showing no difference in osteogenesis between $Rhot1^{fl/fl}$ and $Prrx1^{cre}$ $Rhot1^{fl/fl}$ mice. **i,** qPCR of osteoblast signature gene expression at the mRNA level showing no difference in osteogenesis between $Rhot1^{fl/fl}$ and $Prrx1^{cre}$ $Rhot1^{fl/fl}$ mice (n = 3 per group). **j,** ELISAs of the concentrations of RANKL, OPG and RANKL/OPG in the serum between $Rhot1^{fl/fl}$ and $Prrx1^{cre}$ $Rhot1^{fl/fl}$ mice (n = 7 in $Rhot1^{fl/fl}$ group and n = 6 in $Prrx1^{cre}$ $Rhot1^{fl/fl}$ group). Scale bar, 250 µm. Data are presented as the mean ± s.d., with biologically individual data points shown. P values were determined by Mann-Whitney U test **(c,** Tb.N of **d,** *Tnfrsf11* of **i,** RANKL/OPG of **j)**, unpaired two-tailed Student's t-test **(d, f, i, j)**, unpaired two-tailed Student's t-test with Welch's correction **(***Sp7* of **i)**. Source data are provided as a Source Data file.

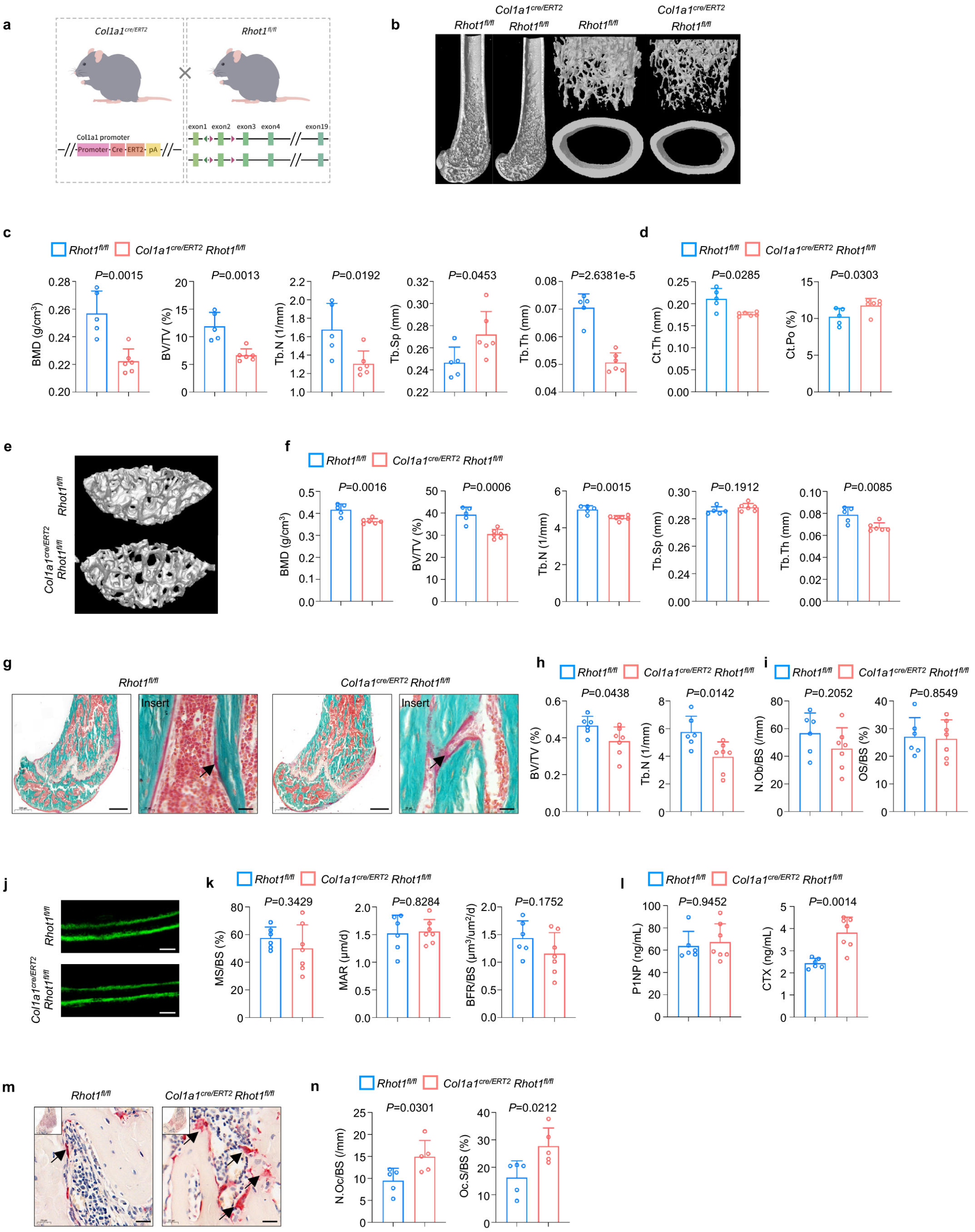

Supplementary Fig. 6 | Impaired mitochondria transfer results in osteoporosis. a, Schematic of the generation of the $Col1a1^{cre/ERT2}$ $Rhot1^{fl/fl}$ transgenic mice line. This figure was created by P.D. and cartoonized by Mr. Zihao Li. **b-d,** Representative µCT reconstructed images of male $Rhot1^{fl/fl}$ and $Col1a1^{cre/ERT2}$ $Rhot1^{fl/fl}$ mouse femurs at 8 weeks **(b)** and trabecular microstructural parameters (BMD, BV/TV, Tb.N, Tb.Sp and Tb.Th) **(c)** and cortical microstructural parameters (Ct.Th and Ct.Po) **(d)** derived from µCT analysis (n = 5 in $Rhot1^{fl/fl}$ group and n = 6 in $Col1a1^{cre/ERT2}$ $Rhot1^{fl/fl}$ group). **e, f,** Representative µCT reconstructed images of male $Rhot1^{fl/fl}$ and $Col1a1^{cre/ERT2}$ $Rhot1^{fl/fl}$ mouse spines at 8 weeks **(e)** and trabecular microstructural parameters (BMD, BV/TV, Tb.N, Tb.Sp and Tb.Th) **(f)** derived from µCT analysis (n = 5 in $Rhot1^{fl/fl}$ group and n

= 6 in *Col1a1*$^{cre/ERT2}$ *Rhot1*$^{fl/fl}$ group). **g-i,** Goldner trichrome staining of male *Rhot1*$^{fl/fl}$ and *Col1a1*$^{cre/ERT2}$ *Rhot1*$^{fl/fl}$ mouse femurs at 8 weeks **(g)** and histomorphometry analysis of BV/TV, Tb.N **(h)**, N.Ob/BS and OS/BS **(i)** (*n* = 6 in *Rhot1*$^{fl/fl}$ group and *n* = 7 in *Col1a1*$^{cre/ERT2}$ *Rhot1*$^{fl/fl}$ group). Scale bar, 500 and 50 μm. **j, k,** Representative images of calcein double labeling of the mineral layers of male *Rhot1*$^{fl/fl}$ and *Col1a1*$^{cre/ERT2}$ *Rhot1*$^{fl/fl}$ mouse femurs at 8 weeks **(j)** and histomorphometric analysis of MS/BS, MAR and BFR/BS **(k)** (*n* = 6 in *Rhot1*$^{fl/fl}$ group and *n* = 7 in *Col1a1*$^{cre/ERT2}$ *Rhot1*$^{fl/fl}$ group). Scale bar, 50 μm. **l,** ELISAs of the concentration of bone formation index P1NP and bone resorption index CTX (*n* = 6 in *Rhot1*$^{fl/fl}$ group and *n* = 7 in *Col1a1*$^{cre/ERT2}$ *Rhot1*$^{fl/fl}$ group). **m, n,** TRAP staining of male *Rhot1*$^{fl/fl}$ and *Col1a1*$^{cre/ERT2}$ *Rhot1*$^{fl/fl}$ mouse femurs at 8 weeks **(m)** and histomorphometric analysis of N.Oc/BS and Oc.S/BS **(n)** (*n* = 5 per group). Scale bar, 50 μm. Data are presented as the mean ± s.d., with biologically individual data points shown. *P* values were determined by unpaired two-tailed Student's *t*-test **(c,** Ct.Th of **d, f, h, i, k, n)**, Mann-Whitney U test **(**Ct.Po of **d,** Tb.N of **h,** P1NP of **l)**, unpaired two-tailed Student's *t*-test with Welch's correction **(**CTX of **l)**. Source data are provided as a Source Data file.

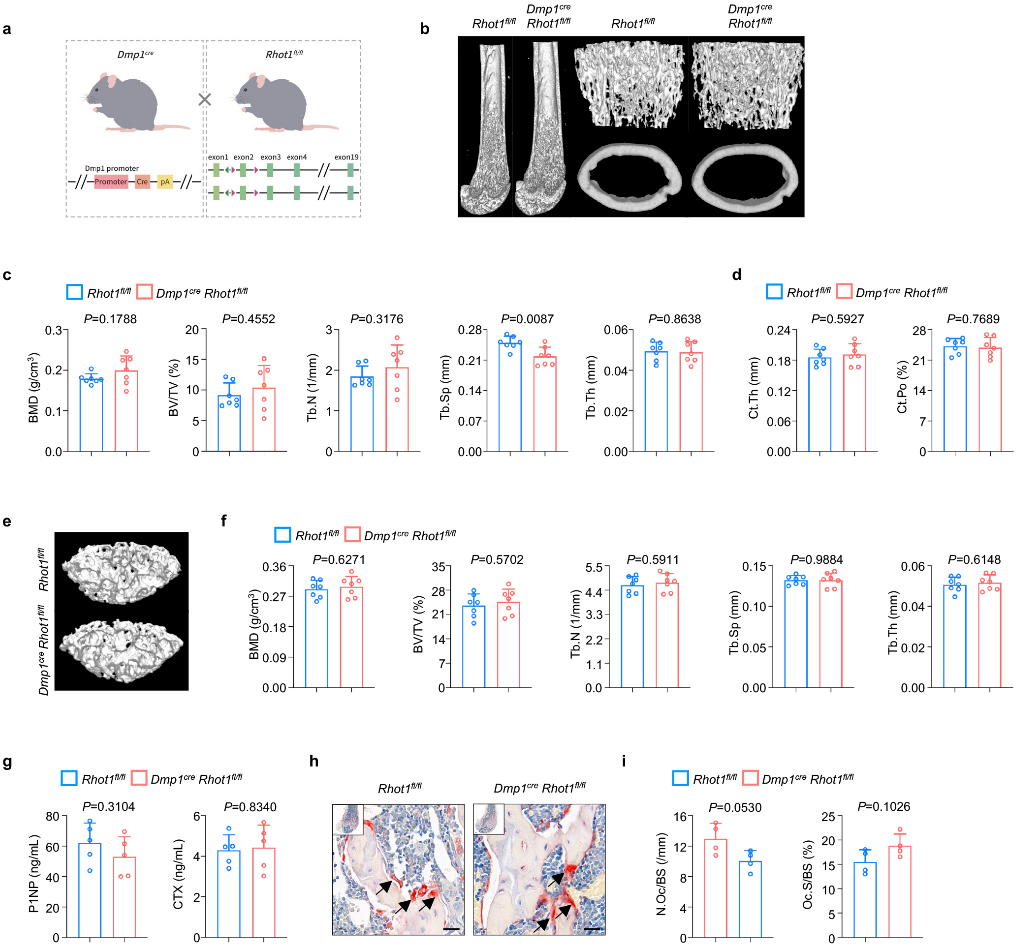

Supplementary Fig. 7 | Osteocytic MIRO1 deficiency did not affect bone mass. a, Schematic of the generation of *Dmp1*^{cre} *Rhot1*$^{fl/fl}$ transgenic mouse line. This figure was created by P.D. and cartoonized by Mr. Zihao Li. **b-d,** Representative µCT reconstructed images of male *Rhot1*$^{fl/fl}$ and *Dmp1*^{cre} *Rhot1*$^{fl/fl}$ mouse femurs at 8 weeks **(b)** and trabecular microstructural parameters (BMD, BV/TV, Tb.N, Tb.Sp and Tb.Th) **(c)** and cortical microstructural parameters (Ct.Th and Ct.Po) **(d)** derived from µCT analysis, demonstrating no difference of bone mass between the *Rhot1*$^{fl/fl}$ and *Dmp1*^{cre} *Rhot1*$^{fl/fl}$ mice (n = 7 per group). **e, f,** Representative µCT reconstructed images of male *Rhot1*$^{fl/fl}$ and *Dmp1*^{cre} *Rhot1*$^{fl/fl}$ mouse spines at 8 weeks **(e)** and trabecular microstructural parameters (BMD, BV/TV, Tb.N, Tb.Sp and Tb.Th) **(f)** derived from µCT analysis (n = 7 per group). **g,** ELISAs of the concentration of bone formation index P1NP and bone resorption index CTX, showing no difference in bone formation and resorption between the *Rhot1*$^{fl/fl}$ and *Dmp1*^{cre} *Rhot1*$^{fl/fl}$ mice (n = 5 per group). **h, i,** TRAP staining of male *Rhot1*$^{fl/fl}$ and *Dmp1*^{cre} *Rhot1*$^{fl/fl}$ mouse femurs at 8 weeks **(h)** and histomorphometric analysis of N.Oc/BS and Oc.S/BS **(i)** (n = 5 per group). Scale bar, 50 µm. Data are presented as the mean ± s.d., with biologically individual data points shown. P values were determined by unpaired two-tailed Student's t-test with Welch's correction (BMD of **c**), and unpaired two-tailed Student's t-test **(c, d, f, g, i)**. Source data are provided as a Source Data file.

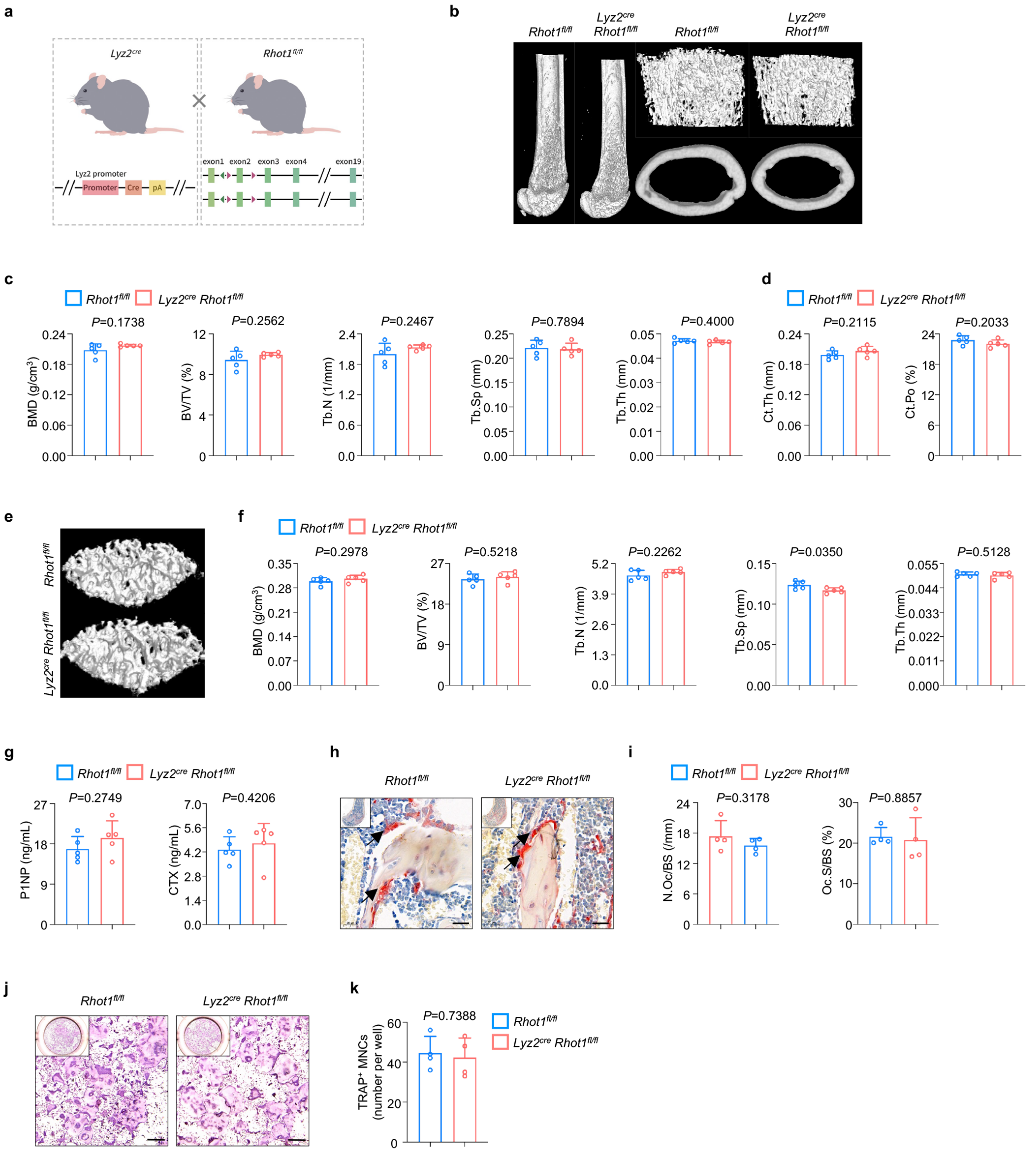

Supplementary Fig. 8 | Osteoclastic MIRO1 deficiency did not affect bone mass. a, Schematic of the generation of the $Lyz2^{cre}$ $Rhot1^{fl/fl}$ transgenic mouse line. This figure was created by P.D. and cartoonized by Mr. Zihao Li. **b-d,** Representative µCT reconstructed images of male $Rhot1^{fl/fl}$ and $Lyz2^{cre}$ $Rhot1^{fl/fl}$ mice femur at 8 weeks **(b)** and trabecular microstructural parameters (BMD, BV/TV, Tb.N, Tb.Sp and Tb.Th) **(c)** and cortical microstructural parameters (Ct.Th and Ct.Po) **(d)** derived from µCT analysis, demonstrating no difference in bone mass between the $Rhot1^{fl/fl}$ and $Lyz2^{cre}$ $Rhot1^{fl/fl}$ mice (n = 5 per group). **e, f,** Representative µCT reconstructed images of male $Rhot1^{fl/fl}$ and $Lyz2^{cre}$ $Rhot1^{fl/fl}$ mouse spines at 8 weeks **(e)** and trabecular microstructural parameters (BMD, BV/TV, Tb.N, Tb.Sp and Tb.Th) **(f)** derived from µCT analysis (n = 5 per group). **g,** ELISAs of the concentration of the bone formation index P1NP and bone resorption index CTX, showing no difference in bone formation and resorption between $Rhot1^{fl/fl}$ and $Lyz2^{cre}$ $Rhot1^{fl/fl}$ mice (n = 5 per group). **h, i,** TRAP staining of male $Rhot1^{fl/fl}$ and $Lyz2^{cre}$ $Rhot1^{fl/fl}$ mouse femurs at 8 weeks **(h)** and histomorphometric analysis of N.Oc/BS and Oc.S/BS **(i)** (n = 4 per group). Scale bar, 50 µm. **j, k,** TRAP

staining of in vitro osteoclastogenesis from *Rhot1*$^{fl/fl}$ and *Lyz2*^{cre} *Rhot1*$^{fl/fl}$ mice **(j)** and quantitative analysis **(k)** of TRAP-positive cells (nucleus > 3) per well ($n = 4$ per group). Scale bar, 250 μm. Data are presented as the mean ± s.d., with biologically individual data points shown. *P* values were determined by unpaired two-tailed Student's *t*-test **(**Tb.Sp, Tb.Th of **c, d, f,** P1NP of **g,** N.Oc/BS of **i, k)**, unpaired two-tailed Student's *t*-test with Welch's correction **(c)**, and Mann-Whitney U test **(**CTX of **g,** Oc.S/BS of **i)**. Source data are provided as a Source Data file.

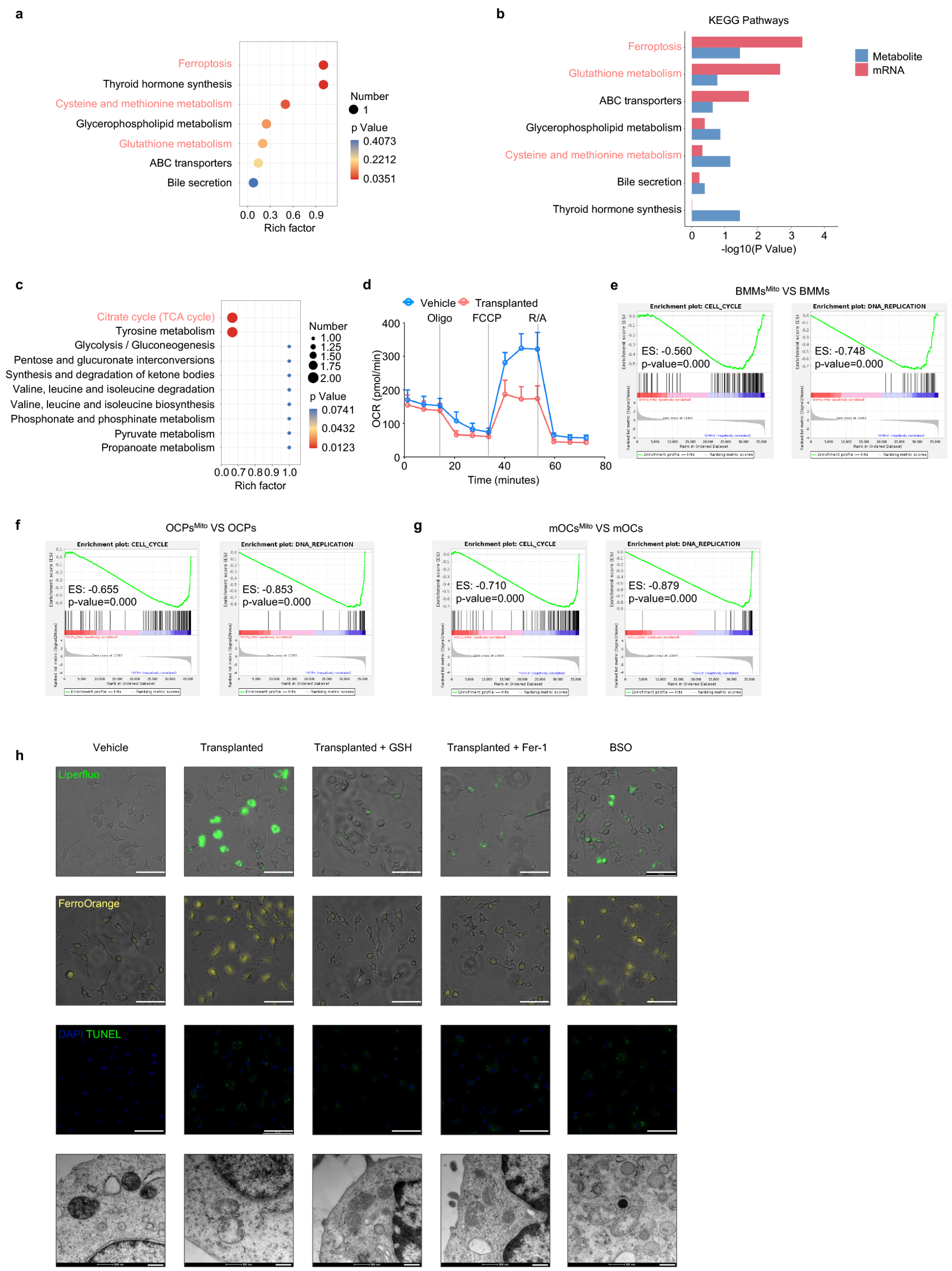

Supplementary Fig. 9 | Osteolineage mitochondria induce osteoclastic ferroptosis. **a,** The top seven enriched KEGG pathways of significantly altered metabolites between mOCs and mOCs^{Mito} (mOCs transplanted with osteolineage cell-derived mitochondria) showing that ferroptosis, cysteine and methionine metabolism, and glutathione metabolism pathways were significantly affected. **b,** Integrated enrichment analysis based on the KEGG pathway showing the enrichment of glutathione metabolism, the ferroptosis pathway and cysteine and methionine metabolism. **c, d,** KEGG pathway enrichment of significantly altered metabolites between OCPs and OCPs^{Mito} (OCPs

transplanted with osteolineage cell-derived mitochondria) illustrating the enrichment of the citrate cycle (TCA cycle) **(c)** and Seahorse metabolic rate analysis showing the significant reduction in the oxygen consumption rate after mitochondria transplantation **(d)** (n = 4 per group). **e-g,** GSEA analysis showing the enrichment of genes associated with cell cycle and DNA replication after mitochondria transplant in BMMs^{Mito} (BMMs transplanted with osteolineage cell-derived mitochondria) **(e)**, OCPs^{Mito} **(f)** and mOCs^{Mito} **(g)**. **h,** OCPs treated with mitochondria transplantation, glutathione depletion (Erastin), mitochondria transplant with glutathione repletion (GSH) and ferroptosis inhibitors (Fer-1). Representative images of Liperfluo staining (scale bar, 62.2 μm), FerroOrange staining (scale bar, 62.2 μm), TUNEL assays (scale bar, 62.2 μm) and TEM (scale bar, 500 nm). Data are presented as the mean ± s.d., with biologically individual data points shown. Source data are provided as a Source Data file.

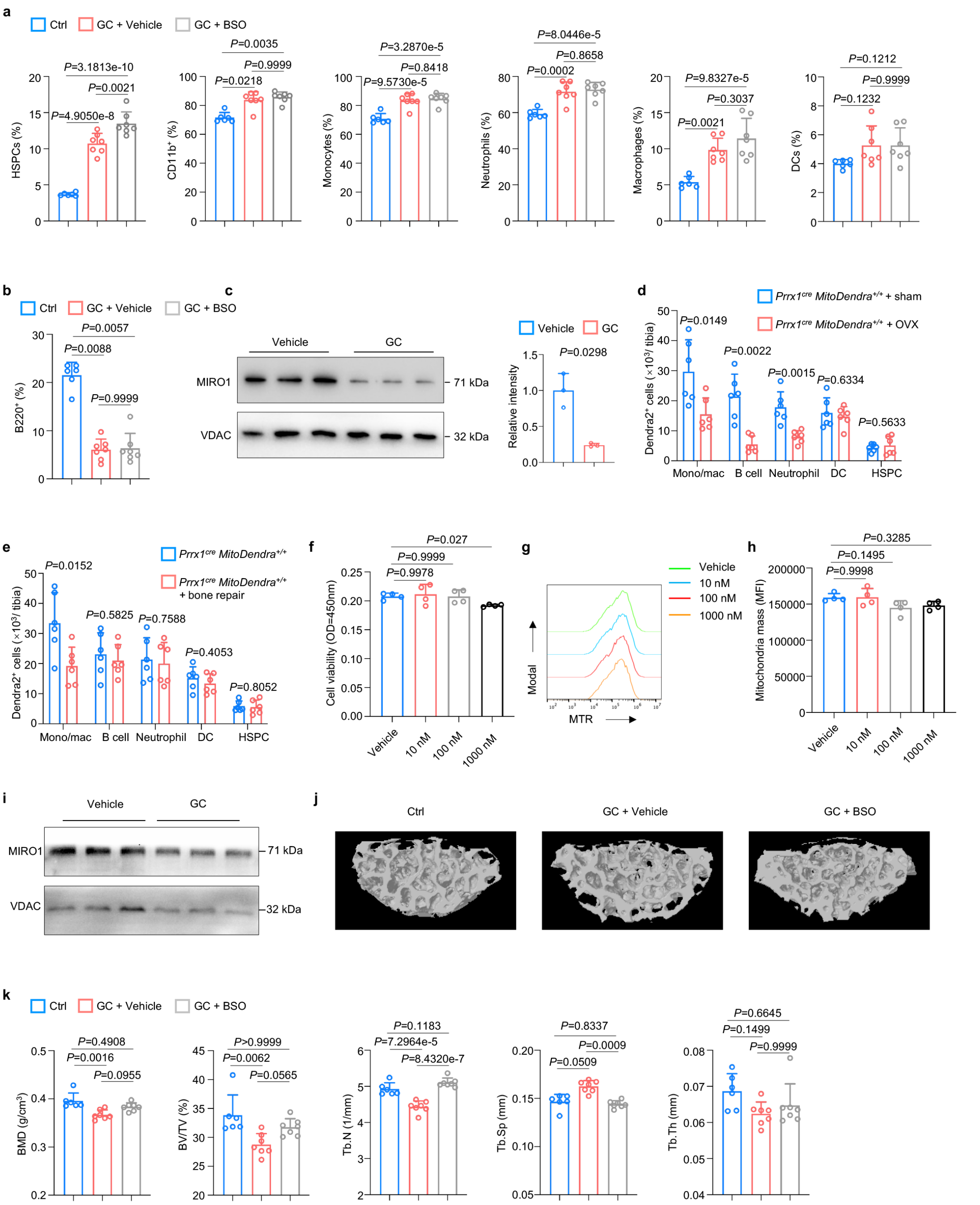

Supplementary Fig. 10 | Osteolineage mitochondria transfer regulates GIOP. a, b, Flow cytometry analysis of the number of HSPCs, myeloid cells **(a)**, and B220$^+$ lymphoid cells **(b)** between Ctrl, GC + Vehicle and GC + BSO group (*n* = 6 in Ctrl group, *n* = 7 in GC + vehicle and GC + BSO group). **c,** Western blot and quantification of sorted osteoprogenitors after GC treatment in vivo (*n* = 3 per group). **d,** Flow cytometry analysis showing decreased mitochondria transfer from osteolineage cells to monocytes/macrophages, B cells, neutrophils in vivo after ovariectomy treatment (*n* = 6 per group). **e,** Flow cytometry analysis showing decreased mitochondria transfer from osteolineage cells to monocytes/macrophages, in vivo during bone repair after bone injury (*n* = 6 per group). **f,** Cell viability of OBs pretreated with GC at different concentrations (10 nM, 100 nM and 1000 nM) for 24 h (*n*=4 per group). **g, h,**

Representative images of flow cytometry **(g)** and analysis **(h)** demonstrating no significant change of mitochondrial biogenesis in OBs after GC pretreatment for 24 h (*n* = 4 per group). **i,** Western blot of OBs pretreated with 100 nM GC for 24 h demonstrating downregulation of MIRO1. **j, k,** Representative μCT reconstructed images of male mouse spines without GC treatment (Ctrl), GC-treated male mice with vehicle treatment (GC + vehicle) and GC-treated male mice with BSO treatment (GC + BSO) for 8 weeks **(e)** and trabecular microstructural parameters (BMD, BV/TV, Tb.N, Tb.Sp and Tb.Th) derived from μCT analysis **(f)** (*n* = 6 in Ctrl group, *n* = 7 in GC + vehicle and GC + BSO group). Data are presented as the mean ± s.d., with biologically individual data points shown. *P* values were determined by ordinary one-way ANOVA with Tukey's multiple comparisons test **(a, h,** Tb.N of **k)**, nonparametric ANOVA with Dunn's multiple comparisons test **(**$CD11b^+$ of **a, b, k)**, Brown-Forsythe and Welch ANOVA tests with Dunnett's T3 multiple comparisons **(f)**, unpaired two-tailed Student's *t*-test **(d, e)**, and unpaired two-tailed Student's *t*-test with Welch's correction **(c,** B cell of **d)**. Source data are provided as a Source Data file.

Supplementary Fig.4a

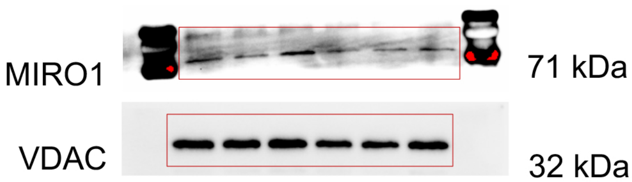

Supplementary Fig.4b

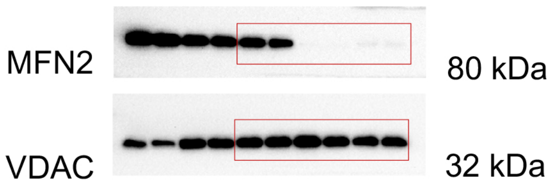

Supplementary Fig.4e

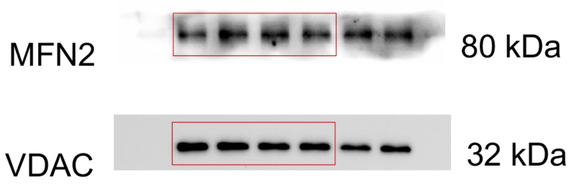

Supplementary Fig.4h

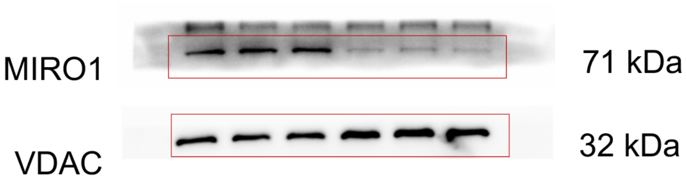

Supplementary Fig.10c

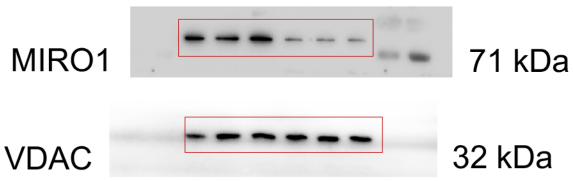

Supplementary Fig.10i

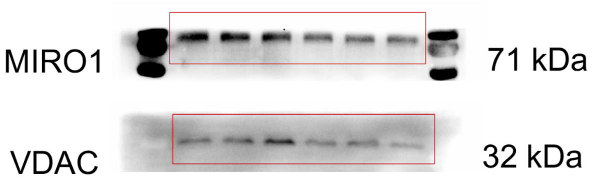

Supplementary Data 1 BMMs_Mito.vs.BMMs_diff.anno

Compound_I	Name	Other_name	Formula	Molecular W	RT [min]	Kegg_ID	KEGG_pathw	HMDB_ID	Lipidmaps_II	SuperClass(
pos-0.961_3	D-(+)-Maltos	--	C12 H22 O1	364.09589	0.961	--	--	HMDB00001	--	Organic oxyg
pos-6.943_5	LPE 20:1	--	C25 H50 N C	507.32925	6.943	--	--	--	LMGP02050	--
pos-6.487_5	C-8 Ceramid	--	C26 H52 N C	505.35022	6.487	--	--	--	--	--
pos-6.257_4	Glycocholic a	Glycocholate	C26 H43 N C	465.30947	6.257	cpd:C01921	map00120 F	HMDB00001	LMST05030	Lipids and lip
pos-3.29_40	YNK	--	C19 H29 N5	406.18329	3.29	--	--	--	--	--
pos-4.231_3	2-Amino-1,3-	--	C18 H39 N C	301.29572	4.231	--	--	HMDB02426	--	Organic nitro
pos-7.103_5	PC (16:0e/2:	--	C26 H54 N C	523.3604	7.103	--	--	--	--	--
pos-6.006_5	PC (16:1e/2:	--	C26 H52 N C	521.34493	6.006	--	--	--	--	--
pos-6.747_4	LPE 18:0	--	C23 H48 N C	481.31371	6.747	--	--	--	LMGP02050	--
pos-6.291_5	PC (14:0e/3:	--	C25 H52 N C	509.34476	6.291	--	--	--	--	--
pos-4.737_2	D-Sphingosi	--	C18 H37 N C	281.26967	4.737	--	--	HMDB00002	--	Organic nitro
pos-5.31_42	ACar 18:1	--	C25 H48 N C	425.34767	5.31	--	--	--	--	--
pos-0.942_1	Creatine	Creatine; alp	C4 H9 N3 O2	131.0686	0.942	cpd:C00300	map00260 (	HMDB00000	--	Organic acid
pos-4.738_3	Sphingosine	--	C18 H37 N C	299.2802	4.738	--	--	--	--	--
pos-1.588_6	L-Glutathion	--	C20 H32 N6	612.14817	1.588	--	--	--	--	--
pos-0.868_1	N6,N6,N6-Tr	N6,N6,N6-Tr	C9 H20 N2 C	188.15118	0.868	cpd:C03793	map00310 L	HMDB00013	--	Organic acid
pos-5.517_4	LPC 15:0	--	C23 H48 N C	481.31368	5.517	--	--	--	LMGP01050	--
pos-5.47_54	PC (18:4e/2:	--	C28 H50 N C	543.32924	5.47	--	--	--	--	--
pos-3.331_2	Ala-Ile	--	C9 H18 N2 C	202.13078	3.331	--	--	HMDB00286	--	Organic acid
pos-6.242_5	PC (18:4e/4:	--	C30 H54 N C	571.36022	6.242	--	--	--	--	--
pos-5.753_5	PC (18:5e/4:	--	C30 H52 N C	569.34462	5.753	--	--	--	--	--
pos-2.881_3	5-Fluoro-2-[(	--	C19 H20 F N	309.16741	2.881	--	--	--	--	--
pos-3.272_4	Hydrocortiso	Cortisol 21-a	C23 H32 O6	404.2182	3.272	cpd:C02821	--	HMDB00000	--	Lipids and lip
pos-5.909_5	PC (18:3e/2:	--	C28 H52 N C	545.34484	5.909	--	--	--	--	--
pos-3.491_2	2-(2-amino-3	--	C14 H20 N2	264.14598	3.491	--	--	--	--	--
pos-5.77_49	PC (14:0e/2:	--	C24 H50 N C	495.32923	5.77	--	--	--	--	--
pos-0.916_9	β-Alanine	beta-Alanine	C3 H7 N O2	89.04711	0.916	cpd:C00099	map00240 F	--	--	--
pos-7.275_3	Bisphenol G	--	C21 H28 O2	329.23648	7.275	--	--	--	--	--
pos-5.246_4	PC (14:1e/2:	--	C24 H48 N C	493.31362	5.246	--	--	--	--	--
pos-7.287_3	2-Arachidon	--	C23 H38 O4	378.27433	7.287	--	--	--	LMGL01010	--
pos-5.737_5	PC (14:1e/3:	--	C25 H50 N C	507.32937	5.737	--	--	--	--	--
pos-3.332_1	Glycyl-L-leu	Glycyl-leucin	C8 H16 N2 C	188.11519	3.332	cpd:C02155	--	HMDB00289	--	Organic acid
pos-3.367_2	2-(2-hydroxy	--	C11 H21 N C	231.14601	3.367	--	--	--	--	--
pos-3.314_2	Gly-Phe	--	C11 H14 N2	222.09938	3.314	--	--	HMDB00288	--	Organic acid
pos-4.901_3	ACar 16:1	--	C23 H44 N C	397.31651	4.901	--	--	--	--	--
pos-5.273_4	LPE 16:1	--	C21 H42 N C	451.2668	5.273	--	--	--	LMGP02050	--

pos-1.714_1	N6-Acetyl-L-	N6-Acetyl-L-	C8 H16 N2 (	188.11501	1.714	cpd:C02727	map00310	L	HMDB00002	--	Organic acid
neg-0.921_3	Trehalose	alpha,alpha-T	C12 H22 O1	342.1163	0.921	cpd:C01083	map00500	S	HMDB00009	--	Organic oxyg
neg-0.87_18	D-(-)-Mannitc	--	C6 H14 O6	182.07881	0.87	--	--		HMDB00007	--	Organic oxyg
neg-3.368_2	Tyrosylalanir	--	C12 H16 N2	252.11111	3.368	--	--		HMDB00290	--	Organic acid
neg-3.304_2	Ala-Leu	--	C9 H18 N2 (	202.13165	3.304	--	--		HMDB02515	--	Organic acid
neg-3.308_2	Thr-Leu	--	C10 H20 N2	232.14223	3.308	--	--		HMDB02590	--	Organic acid
neg-9.849_5	LPI 16:0	--	C25 H49 O1	572.29628	9.849	--	--		--	LMGP06050	--
neg-5.728_4	LysoPE 18:0	--	C23 H48 N (	481.31686	5.728	--	--		--	--	--
neg-1.692_1	Erythronolac	--	C4 H6 O4	118.02644	1.692	--	--		--	--	--
neg-3.468_2	Feruloyl Putr	--	C14 H20 N2	264.14747	3.468	--	--		--	--	--
neg-6.917_6	LPI 20:4	--	C29 H49 O1	620.29637	6.917	--	--		--	LMGP06050	--
neg-3.664_3	Phe-Phe	--	C18 H20 N2	312.14755	3.664	--	--		HMDB00133	--	Organic acid
neg-3.308_1	N-(5-Aminop	--	C7 H16 N2 (	144.1261	3.308	--	--		HMDB00022	--	Organic acid
neg-3.705_3	Bestatin	--	C16 H24 N2	308.17368	3.705	--	--		--	--	--
neg-5.522_5	LPE 20:4	--	C25 H44 N (	501.2856	5.522	--	--		--	LMGP02050	--
neg-6.422_5	LPC 17:0	--	C25 H52 N (	555.35337	6.422	--	--		--	LMGP01050	--
neg-3.515_3	2-[(3S)-1-(Cy	--	C18 H24 F N	301.20029	3.515	--	--		--	--	--
neg-6.644_5	LPE 22:3	--	C27 H50 N (	531.33216	6.644	--	--		--	--	--
neg-6.04_52	LPE 22:4	--	C27 H48 N (	529.31664	6.04	--	--		--	LMGP02050	--
neg-4.287_3	Prostaglandi	Prostaglandi	C20 H32 O6	368.21956	4.287	cpd:C05956	map00590	A	HMDB00032	LMFA03010(	Lipids and lip
neg-7.077_5	LPC 18:0	--	C26 H54 N (	569.36923	7.077	--	--		--	LMGP01050	--
neg-3.293_6	glutathione d	Glutathione (	C20 H32 N6	612.15254	3.293	cpd:C00127	map00480	(	HMDB00033	--	Organic acid
neg-6.201_6	LPC 22:4	--	C30 H54 N (	617.3693	6.201	--	--		--	LMGP01050	--
neg-7.719_3	Arachidonic a	Arachidonate	C20 H32 O2	304.23999	7.719	cpd:C00219	map00590	A	HMDB00010	LMFA01030(	Lipids and lip
neg-4.567_3	Prostaglandi	Alprostadil; F	C20 H34 O5	354.24046	4.567	cpd:C04741	--		HMDB00014	LMFA03010:	Lipids and lip
neg-5.444_5	Lysops 22:6	--	C28 H44 N (	569.27293	5.444	--	--		--	--	--
neg-1.42_11	UDP-N-acety	UDP-N-acety	C17 H27 N3	112.02711	1.42	cpd:C00043	map00520	A	HMDB00002	--	Nucleosides,
neg-5.536_5	LPC 20:4	--	C28 H50 N (	589.33817	5.536	--	--		--	LMGP01050	--
neg-5.707_6	LPC 22:5	--	C30 H52 N (	615.35379	5.707	--	--		--	LMGP01050	--
neg-8.344_2	12,13-EODE	--	C18 H32 O3	296.23485	8.344	--	--		--	--	--
neg-7.08_50	Lysopc 17:0	--	C25 H52 N (	509.34775	7.08	--	--		--	--	--
neg-5.595_5	LPE 22:5	--	C27 H46 N (	527.3011	5.595	--	--		--	LMGP02050	--
neg-5.4_524	LPE 22:6	--	C27 H44 N (	525.28546	5.4	--	--		--	LMGP02050	--

Class(HMDB	SubClass(HI	CATEGORY	MAIN_CLAS	SUB_CLASS	mzCloud_Re	mzVault_Re	MassList_Re	m/z	pos_BMMs	pos_BMMs
Organooxyge	Carbohydrat	--	--	--	Invalid mass	Invalid mass	No results	365.103	7608716.5	10171851
--	--	Glycerophos	Glycerophos	Monoacylgly	No results	Invalid mass	No results	508.33649	1025407.2	1126140.2
--	--	--	--	--	Invalid mass	No results	No results	506.35782	1373652.9	1331874.5
Steroids and	Bile acids, al	Sterols [ST]	Steroid conju	Glycine conju	No results	No results	Full match	466.31674	198517.54	185942.85
--	--	--	--	--	Invalid mass	No results	No results	407.19055	775081.06	1175967.3
Organonitrog	Amines	--	--	--	Invalid mass	Invalid mass	No results	302.30313	601518.4	872406.51
--	--	--	--	--	No results	Invalid mass	No results	524.3678	151702275	172574356
--	--	--	--	--	No results	Invalid mass	No results	522.35242	8639372.9	10592416
--	--	Glycerophos	Glycerophos	Monoacylgly	No results	Invalid mass	No results	482.3208	51554586	64983236
--	--	--	--	--	No results	Invalid mass	No results	510.35217	1676531.3	2028988.8
Organonitrog	Amines	--	--	--	Invalid mass	No results	No results	282.27692	30994901	34664976
--	--	--	--	--	No results	Invalid mass	No results	426.35489	21771034	27237032
Carboxylic a	Amino acids,	--	--	--	Invalid mass	Invalid mass	No results	132.07591	241456592	307870657
--	--	--	--	--	Invalid mass	Invalid mass	No results	300.28723	63534668	72019043
--	--	--	--	--	Invalid mass	Invalid mass	No results	613.15546	192821984	218783428
Carboxylic a	Amino acids,	--	--	--	Invalid mass	Invalid mass	No results	189.15845	2985905.2	4860424.2
--	--	Glycerophos	Glycerophos	Monoacylgly	No results	Invalid mass	No results	482.32104	3550223.8	4956058.6
--	--	--	--	--	No results	Invalid mass	No results	544.33649	3036926.5	3639135.3
Carboxylic a	Amino acids,	--	--	--	No results	Full match	Full match	203.13806	8663734.8	5378354.5
--	--	--	--	--	No results	Invalid mass	No results	572.36755	4542018.5	7390884.1
--	--	--	--	--	No results	Invalid mass	No results	570.35199	5538459.4	8415404.7
--	--	--	--	--	Invalid mass	No results	No results	310.17468	2129674.1	2899483.1
Steroids and	Hydroxysterc	--	--	--	No results	No results	Full match	405.22601	126723.71	217116.8
--	--	--	--	--	No results	Invalid mass	No results	546.35211	3969861.8	4561500.9
--	--	--	--	--	Invalid mass	Invalid mass	No results	265.15335	12865437	9777515.8
--	--	--	--	--	No results	Invalid mass	No results	496.33667	11252675	17140792
--	--	--	--	--	Invalid mass	Invalid mass	No results	90.05437	6221760.1	8443680.3
--	--	--	--	--	Invalid mass	No results	No results	330.24374	999066.82	1721031.3
--	--	--	--	--	No results	Invalid mass	No results	494.32092	696206.33	790511.74
--	--	Glycerolipids	Monoradylgly	Monoacylgly	Invalid mass	Invalid mass	No results	379.28165	4592195.7	9140404.5
--	--	--	--	--	No results	Invalid mass	No results	508.33707	1437046.9	2974434.1
Carboxylic a	Amino acids,	--	--	--	Full match	Full match	Full match	189.12244	3124328.7	4303193.9
--	--	--	--	--	Full match	No results	Full match	232.15331	1027472.5	1279621.6
Carboxylic a	Amino acids,	--	--	--	No results	No results	Full match	223.1066	3242156.9	6285836.4
--	--	--	--	--	No results	Invalid mass	No results	398.32379	4432052.4	5120665.7
--	--	Glycerophos	Glycerophos	Monoacylgly	No results	Invalid mass	No results	452.27393	1592165.9	1494631.4

Carboxylic a	Amino acids,	--	--	--	Invalid mass	Invalid mass	No results	189.12244	6900977.8	5307111.2
Organooxyge	Carbohydrat	--	--	--	No results	No results	Full match	341.10901	2246386.9	2421146
Organooxyge	Carbohydrat	--	--	--	Full match	Full match	Full match	181.07153	3405323.2	3752588.8
Carboxylic a	Amino acids,	--	--	--	Full match	No results	No results	251.10384	220065.79	178097.49
Carboxylic a	Amino acids,	--	--	--	No results	Full match	Full match	201.12436	907063.01	596337.7
Carboxylic a	Amino acids,	--	--	--	No results	No results	Full match	231.13493	341597.88	216267.66
--	--	Glycerophos	Glycerophos	Monoacylgly	No results	Full match	No results	571.28906	478413.32	397381.89
--	--	--	--	--	No results	No results	Full match	480.30942	122543.71	171803.72
--	--	--	--	--	No results	Full match	Full match	117.01916	2932647.8	3562834.1
--	--	--	--	--	No results	No results	Full match	263.14014	781559.46	852656.13
--	--	Glycerophos	Glycerophos	Monoacylgly	No results	Full match	No results	619.2887	3788815.3	2802215.5
Carboxylic a	Amino acids,	--	--	--	No results	No results	Full match	311.14014	206894.14	176884.66
Carboxylic a	Carboxylic a	--	--	--	No results	No results	Full match	143.11879	16007.536	17273.199
--	--	--	--	--	No results	Full match	No results	307.16638	108861.03	144923.94
--	--	Glycerophos	Glycerophos	Monoacylgly	No results	Full match	No results	500.27832	11424887	7771137.8
--	--	Glycerophos	Glycerophos	Monoacylgly	No results	Invalid mass	No results	554.34607	430345.88	395518.79
--	--	--	--	--	Invalid mass	No results	No results	300.19284	145177.44	329176.27
--	--	--	--	--	No results	Full match	No results	530.32495	594081.78	503956
--	--	Glycerophos	Glycerophos	Monoacylgly	No results	Full match	No results	528.30927	9414669.4	7538871.3
Fatty Acyls	Eicosanoids	Fatty Acyls [I	Eicosanoids	Prostaglandi	No results	No results	Full match	367.21228	53208.573	73611.613
--	--	Glycerophos	Glycerophos	Monoacylgly	No results	Invalid mass	No results	568.36182	8357239.3	6351482.3
Carboxylic a	Amino acids,	--	--	--	No results	No results	Full match	611.14526	199148.52	123868.22
--	--	Glycerophos	Glycerophos	Monoacylgly	No results	Invalid mass	No results	616.36145	585550.95	528968.22
Fatty Acyls	Fatty acids a	Fatty Acyls [I	Fatty Acids a	Unsaturated	Full match	Full match	Full match	303.23273	284439.34	469959.85
Fatty Acyls	Eicosanoids	Fatty Acyls [I	Eicosanoids	Prostaglandi	No results	No results	Full match	353.23322	1473074.4	1215889.9
--	--	--	--	--	No results	No results	Full match	568.2652	1015111.7	1033706.8
Pyrimidine n	Pyrimidine n	--	--	--	Invalid mass	No match	No match	111.01985	865748.18	646310.48
--	--	Glycerophos	Glycerophos	Monoacylgly	No results	Invalid mass	No results	588.33087	4130607.9	4234956.2
--	--	Glycerophos	Glycerophos	Monoacylgly	No results	Invalid mass	No results	614.34625	769410.8	540921.25
--	--	--	--	--	No results	No results	Full match	295.22757	26247.311	17388.16
--	--	--	--	--	No results	No results	Full match	508.3399	257644.68	216327.78
--	--	Glycerophos	Glycerophos	Monoacylgly	No results	Full match	No results	526.2937	7086666.6	6155841.4
--	--	Glycerophos	Glycerophos	Monoacylgly	No results	Full match	No results	524.27789	6223109.6	4257742.6

pos BMMs	pos BMMs	pos BMMs	pos BMMs	pos BMMs	pos BMMs	FC	log2FC	Pvalue	ROC	VIP
11687831	12137390	64268.627	84113.436	93501.936	88944.885	125.76226	6.9745552	6.40E-08	1	4.3425036
1056630.3	992474.63	480895.68	591457.71	469642.4	508682.22	2.0484212	1.0345124	0.000121	1	1.4349074
2351818.7	1816343.4	436785.29	359688.44	354636.02	284796.84	4.7870032	2.2591228	0.0001619	1	2.8578516
291383.74	183987.37	37736.342	45879.391	70737.587	40060.152	4.4226951	2.1449258	0.0002188	1	3.0332346
1106913.4	842320.78	281652.41	298580.33	357216.34	317757.43	3.1072836	1.6356539	0.0003605	1	2.3001737
834710.82	706176.77	332647.3	325810.23	424382.41	356000.27	2.0953074	1.0671619	0.0006357	1	1.0058065
202264881	177201432	69404285	104059808	82718536	75390541	2.1224363	1.0857213	0.0006484	1	1.8181944
12919509	8755597	5840822.7	4189482.4	4273447.9	4342837.6	2.1938002	1.1334322	0.0008159	1	1.7406063
69017006	83152065	31714180	26721955	19532666	30903461	2.4680932	1.3033969	0.0009351	1	2.4063194
2254446.3	2108045.1	1157282.6	1277386.9	1319724.6	993992.62	1.6991058	0.7647757	0.0010572	1	1.6769286
44127115	50908607	14839060	19401099	14085172	18698923	2.3975738	1.2615752	0.0011393	1	2.3327575
33874080	36091542	6752267.7	11415274	12242378	11274785	2.8541329	1.5130525	0.0011772	1	1.3707724
339637896	370187172	109145638	96220243	107213082	173132728	2.5923863	1.3742807	0.0013428	1	2.4145951
86009421	110707202	29330521	36840193	29019362	36584992	2.5214962	1.33428	0.0015426	1	2.4819816
258515231	305334609	64969647	88766015	112419386	117853774	2.5401897	1.3449362	0.0018632	1	1.8637445
4764656.8	3582315.6	1720234.9	1590352.6	1555945	2069342.3	2.3347166	1.2232474	0.0020164	1	1.0877382
4905335.4	5029520.4	2359832.1	2486759.9	2151917	2452788.5	1.9511753	0.9643434	0.0020632	1	1.8354311
4952335.8	5456031.8	1885741.4	1835661	1272610.6	1822760.6	2.506234	1.3255211	0.002251	1	1.4233442
7074911.3	5287114.4	17391496	18623163	42899000	31600960	0.2389197	-2.0654025	0.0031429	1	2.7302816
10664509	9514480.1	2526029.6	2596362.3	2145258.8	1855663.1	3.5197618	1.8154778	0.0040541	1	1.8776168
8998157	7663128.6	3784249.9	3627955.6	2029392.4	3598130.4	2.3478365	1.2313319	0.0041235	1	2.1892398
4577899.9	4909983.6	1065494.7	807247.71	1034272.1	1659012.2	3.1793595	1.6687362	0.0044754	1	1.2692501
186086.55	327079.81	98896.035	58134.311	75781.247	65902.149	2.8689904	1.5205432	0.0071106	1	1.195154
4019432.7	6175376.7	3419438.7	1598579.8	1889499.6	2115783.7	2.0753126	1.0533287	0.0109674	1	2.097996
11026397	7904828.7	15749583	16663470	30163833	20775599	0.4987755	-1.0035374	0.0114633	1	1.2967143
23377967	24513208	6848196.6	10544534	7166909.9	9106384.1	2.2659237	1.1800993	0.0135678	1	2.1174657
10748978	10061721	5035026.8	5077693.8	4507519.2	5722711.5	1.7439033	0.80232	0.0154738	1	1.1639025
2035317.5	2003773.3	1125252.3	545839.7	647832	834171.16	2.1436679	1.1000814	0.0160345	0.9375	1.3820976
994469.1	1273751.9	559850.85	627996.81	321668.35	489874.97	1.8780414	0.9092289	0.0187276	1	1.3272678
10861544	14730086	3147546.6	3176322.7	4217192.4	3433924	2.8139013	1.4925717	0.025984	1	2.8085733
3028332.9	2848836.4	1192015.7	1153779.5	1145599.8	1632995.4	2.0077804	1.0056015	0.026077	0.9375	1.7660448
5367273.3	5347467.9	9861918.2	5959306.6	6495582.9	7351584.4	0.6115014	-0.7095723	0.026416	1	1.0427051
1428946.6	1869545.2	1645781.5	2474513.3	2208199.5	2663593.4	0.6233909	-0.6817909	0.0268241	0.9375	1.4808162
7346305.7	8041704.2	3456246.1	2785851.7	2296416.3	2708846.5	2.2152756	1.1474862	0.0280466	0.9375	1.0710172
6801782	8761541.7	1047032.6	2705945.2	3774186	2671352	2.4627154	1.3002499	0.0306843	1	1.1646053
1731725.2	3547547.7	1061077.1	967529.6	924509.8	921799.04	2.1590329	1.1103852	0.0374973	1	1.5280201

5217144.7	3060280.6	11135621	11395770	5557890.8	13943906	0.4873652	-1.0369247	0.0375795	0.9375	1.2178874
2573721.6	2768718	133819.24	126171.82	134844.19	130486	19.054955	4.2520943	1.06E-06	1	2.8308961
4712413.8	4677908.3	258651.02	232296.31	227224.07	216363.8	17.707449	4.1462844	3.24E-06	1	3.137848
210024.65	137272.09	827367.02	577788.67	827968.91	557685.45	0.2671124	-1.904481	0.0001324	1	2.0382684
671574.1	443626.35	2626366	2628560.8	3825225.2	2885971.6	0.2188345	-2.1920876	0.0003222	1	1.9416301
238643.58	161448.08	1045180.8	816494.19	1222089.4	855816.17	0.2431622	-2.0400089	0.0005401	1	1.9025014
506457.22	394201.02	225920.25	186162.56	150351.48	144726.89	2.5120913	1.3288889	0.0005978	1	1.4566256
170926.65	125683.89	70278.319	48763.229	39190.179	43303.308	2.932284	1.5520249	0.0006708	1	1.9030092
3314150.3	3742908.6	1981692.1	2055872	2105129.7	2464611.1	1.5745394	0.6549298	0.00075	1	1.1429785
665060.64	521091.41	1611336.2	1478831.6	1873666.8	1700272.6	0.4232176	-1.2405284	0.0015065	1	1.3349344
3758465.1	2285402.8	1507179.3	1051829.7	730570.04	724245.75	3.1478451	1.6543646	0.0021645	1	2.4177621
163307.22	170650.07	474773.37	327003.02	563321.39	371770.8	0.4132357	-1.2749632	0.0027321	1	1.3876143
15909.067	28302.741	62833.739	32186.678	49629.713	44691.972	0.4092726	-1.2888659	0.0037763	1	1.5394592
129605.71	107606.61	12479.134	31868.817	29840.629	33196.315	4.5723125	2.192924	0.004323	1	2.0228908
8308114.8	7172813.1	5827256.4	3143294	3300957.9	3956390.3	2.1368726	1.0955009	0.0052899	1	1.3335883
534344.62	463636.32	307878.84	255741.07	178762.82	198271.97	1.9389108	0.9552465	0.0059333	1	1.2518777
306482.18	240886.28	766989.93	559470.16	609264.99	786928.79	0.375267	-1.4140106	0.0066272	1	1.103107
597645.79	583973.04	234567	90283.691	74054.29	99644.491	4.5725785	2.1930079	0.0069348	1	3.144857
7904867.2	7275176.3	5529336.9	3208533.9	2497808.7	3214809.4	2.2237022	1.1529636	0.0110246	1	1.4455481
51063.809	48622.334	43099.416	22568.45	26707.866	32846.523	1.8088344	0.8550604	0.0139775	1	1.447459
8043904.2	8307548.4	4992840.2	3149270.7	1853134	2656008.8	2.4551064	1.2957856	0.0144557	1	1.63555
279693.21	160137.63	79832.089	92447.045	81824.223	72440.854	2.3361234	1.2241165	0.0146775	1	1.9663746
565808.82	662811.38	393540.49	214171.36	153179.19	176260.92	2.5002769	1.3220879	0.0154342	1	1.2260041
437277.39	777231.76	185555.45	154633.22	202839.37	199913.38	2.6501529	1.4060756	0.016891	1	1.6716011
1189083.1	976767.35	903112.59	339141.55	461471.85	388715.33	2.3201677	1.2142291	0.0185765	1	2.1778649
857779.85	1096428.3	789359.29	497660.49	367709.8	398183.19	1.9499253	0.9634189	0.0208578	1	1.161193
758649.22	637913.97	973327.91	892752.79	984710.91	902935.04	0.7748625	-0.3679878	0.0301613	1	1.3872934
4630819	4479364.2	3658316.6	1625674.2	1298854.3	1877892.5	2.0655111	1.0464988	0.0354684	1	1.0183237
913983.78	949327.42	663036.7	292128.33	275071.19	443565.58	1.8960687	0.9230113	0.0367711	0.9375	1.2095673
37222.822	29850.583	16966.604	14594.676	13965.677	17543.111	1.7553315	0.8117435	0.0387476	0.9375	1.6037155
256267.79	258531.86	202629.09	121002.77	67004.513	99612.337	2.0168786	1.0121242	0.0404253	1	1.3680079
6296626.2	5572180.4	5954770.8	3463434.3	2782122.5	3451653.3	1.6043538	0.6819923	0.045966	0.9375	1.2054599
4541502.9	4272731.7	4676360.6	2887489.5	2529628.9	2459209.7	1.5371278	0.6202371	0.0489328	0.8125	1.093485

Up.Down
up
up
up
up
up
up
up
up
up
up
up
up
up
up
up
up
up
up
down
up
up
up
up
up
down
up
up
up
up
up
up
down
down
up
up
up

down
up
up
down
down
down
up
up
up
down
up
down
down
up
up
up
down
up
up
up
up
up
up
up
up
up
down
up
up
up
up
up
up

Supplementary Data 2 mOCs_Mito.vs.mOCs_diff.anno

Compound_I	Name	Other_name	Formula	Molecular W	RT [min]	Kegg_ID	KEGG_pathw	HMDB_ID	Lipidmaps_II	SuperClass(I
neg-0.93_14	D-Ribose	D-Ribose	C5 H10 O5	150.05258	0.93	cpd:C00121	map00030 F	HMDB00002	--	Organic oxyg
neg-0.864_1	D-Gluconic a	D-Gluconic a	C6 H12 O7	196.05802	0.864	cpd:C00257	map00030 F	HMDB03037	--	Organic acid
neg-7.344_2	16-Hydroxyh	--	C16 H32 O3	272.23505	7.344	--	--	HMDB00062	--	Lipids and lip
neg-3.261_3	N6-Succinyl	--	C14 H17 N5	383.1082	3.261	--	--	HMDB02553	--	Organic acid
neg-3.821_2	2-Methylbuty	--	C12 H23 N (	245.16247	3.821	--	--	HMDB00003	LMFA07070(	Lipids and lip
neg-1.441_1	Glutaconic a	(E)-Glutacon	C5 H6 O4	130.0265	1.441	cpd:C02214	--	HMDB00006	LMFA01170:	Organic acid
neg-0.866_1	N-Ethylglycir	N-Ethylglycir	C4 H9 N O2	103.06311	0.866	cpd:C11735	--	HMDB00419	--	Organic acid
pos-1.461_2	Tris(2-carbo›	--	C9 H15 O6 F	250.06081	1.461	--	--	HMDB02470	--	Organic acid
pos-3.133_3	MPK	--	C16 H30 N4	356.18565	3.133	--	--	HMDB00342	--	Organic oxyg
pos-0.952_1	Triethanolam	Triethanolam	C6 H15 N O:	149.10419	0.952	cpd:C06771	map00564 (	HMDB00325	--	Organic nitro
pos-1.464_3	L-Glutathion	--	C10 H17 N3	307.08184	1.464	--	--	--	--	--
pos-3.387_2	LysoPC 20:2	--	C28 H54 N (	547.36475	3.387	--	--	--	--	--
pos-4.192_1	Diethyl phos	--	C4 H11 O4 F	154.03831	4.192	--	--	HMDB00122	--	Organic acid
pos-1.468_3	8-Bromogua	--	C10 H12 Br	361.00073	1.468	--	--	--	--	--
pos-1.472_3	Glutathione	Glutathione;	C10 H17 N3	307.08354	1.472	cpd:C00051	map00270 (	HMDB00001	--	Organic acid

Class(HMDB	SubClass(HI	CATEGORY	MAIN_CLAS	SUB_CLASS	mzCloud_Re	mzVault_Re:	MassList_Re	m/z	neg_mOCs_	neg_mOCs_
Organooxyge	Carbohydrate	--	--	--	No results	No results	Full match	149.04529	360783.02	527163.57
Hydroxy acid	Medium-chai	--	--	--	No results	No results	Full match	195.05084	425674.64	804157.23
Fatty Acyls	Fatty acids a	--	--	--	Full match	Full match	No results	271.22781	6263366.2	6351638.8
Carboxylic ac	Dicarboxylic	--	--	--	No results	No results	Full match	382.1011	81295.26	74784.422
Fatty Acyls	Fatty acid es	Fatty Acyls [I	Fatty esters [	Fatty acyl ca	No results	No results	Full match	244.15515	17596.512	33814.472
Carboxylic ac	Dicarboxylic	Fatty Acyls [I	Fatty Acids a	Dicarboxylic	No results	Full match	Full match	129.01927	3699552.6	2707129.6
Carboxylic ac	Amino acids,	--	--	--	No results	Full match	Full match	102.05589	824110.41	1084639.5
Carboxylic ac	Tricarboxylic	--	--	--	No results	No results	Full match	251.06821	2846447.1	1415714
Organooxyge	Carbonyl cor	--	--	--	Invalid mass	No results	No results	357.19354	560509.26	679961.73
Organonitrog	Amines	--	--	--	Invalid mass	Invalid mass	No results	150.11153	5986929.5	9931447.6
--	--	--	--	--	Invalid mass	Invalid mass	No results	308.08917	112685602	29152389
--	--	--	--	--	No results	No results	Full match	274.68961	1897610	1306314.8
Organic phos	Phosphate e	--	--	--	Invalid mass	Invalid mass	No results	155.04552	58591558	220104070
--	--	--	--	--	No results	No results	Full match	362.00812	420350.22	226256.98
Carboxylic ac	Amino acids,	--	--	--	No results	No results	Full match	346.04678	336236.82	57297.515

neg_mOCs	neg_mOCs	neg_mOCs	neg_mOCs	neg_mOCs	neg_mOCs	FC	log2FC	Pvalue	ROC	VIP
1008398.6	192452.51	4601092.6	2684609.6	2993297.9	6477629.6	0.124655	-3.0039874	0.0031004	1	3.513779
1823205.7	572244.12	3660158.2	2265284.2	2308813.4	5678233.1	0.2605775	-1.9402155	0.0119126	1	1.7847633
8866124.7	3681900.3	59264390	12368430	20419733	26319966	0.2125749	-2.2339566	0.0139767	1	2.1725199
172936.99	58176.17	290611.38	130510.57	207792.57	393923.18	0.3785477	-1.4014531	0.0257713	0.9375	1.064793
47193.232	27930.468	52719.505	68854.617	90025.284	42288.517	0.498388	-1.0046589	0.0361412	0.9375	1.5361278
2010268.1	5294851.6	1607483.9	2360938.1	1263909.3	1720785.5	1.9720367	0.9796864	0.0472987	0.9375	1.4182977
2167910.8	985294.5	3226563.7	1619670.2	1846531.9	2881125.9	0.5287249	-0.9194107	0.0499348	0.875	1.1495449
2998413.8	2599821.2	4287354.7	5020381.4	5254186	4142698.7	0.5271637	-0.9236772	0.0246238	1	1.6217415
366560.64	391542.3	175164.56	198372.51	309237.43	78869.533	2.6240262	1.3917821	0.0264464	1	1.6853279
23386549	8457312.4	37712247	20773100	27807207	23496691	0.4350357	-1.2007944	0.0391204	0.9375	2.3790287
173735923	91784399	442257949	153564189	301607244	349039767	0.3268098	-1.6134769	0.0402408	0.9375	2.2006802
744622.92	1568093	624738.1	629538.64	832242.05	690588.53	1.9864701	0.9902071	0.0449687	0.9375	2.1359338
61617153	45537128	23912509	18379873	36514228	27589056	3.6265566	1.8586004	0.0454534	1	1.8722787
517184.93	345551.55	1309369.8	448275.32	772640.81	655263.41	0.4738095	-1.0776208	0.0472658	0.9375	1.8211781
721890.9	372101.43	3436160.7	476456.07	1700089.9	1812682.3	0.2003298	-2.3195511	0.0477205	0.9375	2.0353691

Up.Down
down
down
down
down
down
up
down
down
up
down
down
up
up
down
down

Supplementary Data 3 OCPs_Mito.vs.OCPs_diff.anno

Compound I	Name	Other name	Formula	Molecular W	RT [min]	Kegg ID	KEGG pathw	HMDB ID	Lipidmaps II	SuperClass(
pos-0.961_3	D-(+)-Maltos	--	C12 H22 O1	364.09589	0.961	--	--	HMDB00001	--	Organic oxyg
pos-1.623_1	3-Methylade	3-Methylade	C6 H7 N5	149.06918	1.623	cpd:C00913	--	HMDB00116	--	Organoheter
pos-3.331_2	Ala-Ile	--	C9 H18 N2 C	202.13078	3.331	--	--	HMDB00286	--	Organic acid
pos-1.714_1	N6-Acetyl-L-	N6-Acetyl-L-	C8 H16 N2 C	188.11501	1.714	cpd:C02727	map00310 L	HMDB00002	--	Organic acid
pos-3.625_4	Taurolithoch	Taurolithoch	C26 H45 N C	483.30327	3.625	cpd:C02592	--	HMDB00007	LMST05040	Lipids and lip
pos-7.275_3	Bisphenol G	--	C21 H28 O2	329.23648	7.275	--	--	--	--	--
pos-3.47_41	RMK	--	C17 H35 N7	415.24091	3.47	--	--	--	--	--
pos-3.42_37	ILK	--	C18 H36 N4	372.27166	3.42	--	--	--	--	--
pos-3.299_3	Fmoc-L-Isole	--	C21 H23 N C	353.16321	3.299	--	--	--	--	--
pos-3.391_2	5'-S-Methyl-5	--	C11 H15 N5	297.088	3.391	--	--	HMDB00011	--	Nucleosides,
neg-0.87_18	D-(-)-Mannito	--	C6 H14 O6	182.07881	0.87	--	--	HMDB00007	--	Organic oxyg
neg-0.921_3	Trehalose	alpha,alpha-T	C12 H22 O1	342.1163	0.921	cpd:C01083	map00500 S	HMDB00009	--	Organic oxyg
neg-3.705_3	Bestatin	--	C16 H24 N2	308.17368	3.705	--	--	--	--	--
neg-1.692_1	Erythronolac	--	C4 H6 O4	118.02644	1.692	--	--	--	--	--
neg-1.925_1	3-Hydroxy-3-	--	C6 H10 O5	162.05265	1.925	--	--	HMDB00003	--	Lipids and lip
neg-3.293_1	Methylsuccir	--	C5 H8 O4	132.04212	3.293	--	--	HMDB00018	LMFA01170	Lipids and lip
neg-0.936_1	N-Acetylalan	--	C5 H9 N O3	131.05805	0.936	--	--	HMDB02550	--	Organic acid
neg-0.927_1	acetoacetate	Acetoacetate	C4 H6 O3	102.03154	0.927	cpd:C00164	map00072 S	HMDB03042	--	Organic acid
neg-1.743_1	Pyruvic acid	Pyruvate; Py	C3 H4 O3	148.03695	1.743	cpd:C00022	map00010 G	HMDB00002	LMFA01060	Organic acid
neg-0.933_1	cis-Aconitic a	cis-Aconitate	C6 H6 O6	174.01609	0.933	cpd:C00417	map00020 C	HMDB00000	--	Organic acid
neg-3.308_1	N-(5-Aminop	--	C7 H16 N2 C	144.1261	3.308	--	--	HMDB00022	--	Organic acid
neg-3.368_2	Tyrosylalanir	--	C12 H16 N2	252.11111	3.368	--	--	HMDB00290	--	Organic acid
neg-3.76_15	3,3-Dimethyl	--	C7 H12 O4	160.07336	3.76	--	--	HMDB00024	--	Lipids and lip

Class(HMDB	SubClass(HN	CATEGORY	MAIN_CLAS	SUB_CLASS	mzCloud_Re	mzVault_Res	MassList_Re	m/z	pos_OCPs_Mitc
Organooxyge	Carbohydrate	--	--	--	Invalid mass	Invalid mass	No results	365.103	15351600.36
Imidazopyrin	Purines and	--	--	--	Invalid mass	No results	No results	150.0764	2581074.034
Carboxylic ac	Amino acids,	--	--	--	No results	Full match	Full match	203.13806	20860495.64
Carboxylic ac	Amino acids,	--	--	--	Invalid mass	Invalid mass	No results	189.12244	8976831.286
Steroids and	Bile acids, al	Sterols [ST]	Steroid conju	Taurine conj	No results	No results	Full match	484.31049	580167.3908
--	--	--	--	--	Invalid mass	No results	No results	330.24374	18591904.57
--	--	--	--	--	Invalid mass	No results	No results	416.24832	891275.6135
--	--	--	--	--	Invalid mass	No results	No results	373.27893	1564314.601
--	--	--	--	--	No results	No results	Full match	354.17117	903798.3387
5'-deoxyribor	5'-deoxy-5'-th	--	--	--	Invalid mass	Invalid mass	No results	298.09503	6158494.503
Organooxyge	Carbohydrate	--	--	--	Full match	Full match	Full match	181.07153	10566756.14
Organooxyge	Carbohydrate	--	--	--	No results	No results	Full match	341.10901	10461920.05
--	--	--	--	--	No results	Full match	No results	307.16638	433841.689
--	--	--	--	--	No results	Full match	Full match	117.01916	9559745.518
Fatty Acyls	Fatty acids a	--	--	--	No results	Full match	No results	161.0453	2794399.505
Fatty Acyls	Fatty acids a	Fatty Acyls [I	Fatty Acids a	Dicarboxylic	Full match	Partial match	Full match	131.03476	17026008.65
Carboxylic ac	Amino acids,	--	--	--	No results	Full match	Full match	130.05072	7813941.803
Keto acids an	Short-chain k	--	--	--	No results	No results	Full match	101.02426	2228176.002
Keto acids an	Alpha-keto a	Fatty Acyls [I	Fatty Acids a	Oxo fatty aci	No results	Invalid mass	No match	147.02968	1153128.619
Carboxylic ac	Tricarboxylic	--	--	--	No results	No results	Full match	173.00882	627224.5132
Carboxylic ac	Carboxylic ac	--	--	--	No results	No results	Full match	143.11879	202798.2081
Carboxylic ac	Amino acids,	--	--	--	Full match	No results	No results	251.10384	1253016.88
Fatty Acyls	Fatty acids a	--	--	--	Full match	Not the top h	Full match	159.06602	10770497.16

pos_OCPs_Mitc	pos_OCPs_Mitc	pos_OCPs_Mitc	pos_OCPs_1	pos_OCPs_2	pos_OCPs_3	pos_OCPs_4	FC	log2FC
4922748.709	17987968.06	16708462.92	210444.9111	59243.33855	274040.2373	51985.564	92.277125	6.5279012
236606.0625	735925.7096	2329733.473	112579.8113	84383.34337	155362.384	59821.743	14.274847	3.8354034
10163170.84	2771787.451	19081772.43	5450103.963	835723.6008	1419637.516	1752688.5	5.59065	2.483016
3660632.696	1333412.997	8747912.965	2292187.953	460831.8912	540690.7978	1063325.5	5.2142762	2.382467
497353.0083	440809.7456	613175.7525	241028.344	193742.6144	462270.8403	167763.93	2.0017791	1.0012828
3028034.313	10602316.23	6744715.801	3925470.566	2588974.777	2072830.432	1326520.6	3.9305801	1.9747423
290634.2465	222169.964	765932.8943	248751.9048	108859.0396	210055.3037	79838.899	3.3513444	1.7447399
1273352.854	350313.9704	1445449.928	478129.8133	142866.5655	322031.4889	441406.98	3.3468035	1.7427838
300570.9756	258522.4287	1043859.31	229684.0249	128768.9357	267075.0495	84312.648	3.5314278	1.8202516
3191776.329	7247348.355	7753882.293	11751488.86	16141339.61	13298549.98	6745477.3	0.5079912	-0.9771246
4795887.766	12823628.59	7140606.369	418288.1174	608321.5818	346647.991	1010836.5	14.817736	3.8892532
2899605.178	12685537.71	7616041.543	239280.0107	141497.828	229055.751	150031.67	44.301413	5.4692808
54690.50503	538614.4299	489035.9456	12358.98425	18859.26035	17477.09215	23333.836	21.049562	4.3957183
2993454.092	6508250.418	11431670.43	1810756.055	1075610.594	3377029.795	1330124.6	4.0156761	2.0056429
1487498.959	3140358.079	2354015.029	1352679.539	944637.8048	1282191.997	1129439.8	2.0761047	1.0538792
3465210.2	19329018.83	12621964.63	2580177.381	982269.7058	4542469.512	1043062.7	5.7326542	2.5192033
382274.2637	9459903.273	5866232.692	171705.0055	106632.6171	119686.1898	147061.78	43.153502	5.4314057
397683.8874	2288694.939	2073707.065	605976.7449	217848.4419	163710.3722	508658.17	4.6706932	2.2236367
336897.8339	1535360.995	1145775.827	386158.0037	197622.5587	396043.6966	269396.9	3.3390111	1.7394209
261706.8159	716379.2189	435810.3433	267584.5663	178911.2753	200812.9356	312563.2	2.1264512	1.0884477
71476.30905	43225.35055	142747.9716	78213.21826	17817.65444	26913.74279	14433.395	3.3502293	1.7442598
658821.6564	93167.97594	997078.6569	214206.102	28024.69459	108375.3783	71220.176	7.1168744	2.8312438
1425310.509	17252589.76	11537582.53	1465693.505	947830.634	1885045.11	1079681.5	7.6206897	2.9299216

Pvalue	ROC	VIP	Up.Down
0.000194	1	2.7565395	up
0.0191765	1	1.879533	up
0.0310618	0.9375	2.1432864	up
0.0336249	0.9375	1.7510376	up
0.0356502	0.9375	1.2045886	up
0.0395576	0.9375	1.3899621	up
0.0424046	0.9375	1.1993059	up
0.0433333	0.875	1.2553015	up
0.0434088	0.9375	1.1130223	up
0.0483347	0.875	2.0496813	down
0.0001552	1	2.1934139	up
0.0004721	1	2.5788581	up
0.0123454	1	2.4736567	up
0.0128281	0.9375	2.4645088	up
0.0156884	1	1.6075122	up
0.0169639	0.9375	2.6272575	up
0.0210482	1	2.7585219	up
0.0344307	0.875	1.8288611	up
0.0379782	0.875	2.0662779	up
0.0431951	0.875	1.2696172	up
0.0469366	0.875	1.5544576	up
0.0479766	0.875	2.0555247	up
0.047992	0.875	2.6188118	up
