# Supplementary material for: Mitochondria from osteolineage cells regulate myeloid cell-mediated bone resorption
Source: Nat Commun. 2024 Jun 14;15:5094. doi: 10.1038/s41467-024-49159-3 (PMC11178781; doi:10.1038/s41467-024-49159-3)
Supplement: Supplementary file 2 — Description of Additional Supplementary Files [file 41467_2024_49159_MOESM2_ESM.pdf]

Description of Additional Supplementary Files

Supplementary Data 1: The significantly different metabolites between BMMs and BMMs^{Mito}. Untargeted metabolomics was performed between BMMs and BMMs^{Mito} (BMMs transplanted with osteolineage cell-derived mitochondria) and significantly different metabolites were annotated. The metabolites with VIP > 1 and P-value < 0.05 and fold change ≥2 or FC≤0.5 were considered to be differential metabolites.

Supplementary Data 2: The significantly different metabolites between OCPs and OCPs^{Mito}. Untargeted metabolomics was performed between OCPs and OCPs^{Mito} (OCPs transplanted with osteolineage cell-derived mitochondria) and significantly different metabolites were annotated. The metabolites with VIP > 1 and P-value < 0.05 and fold change ≥2 or FC≤0.5 were considered to be differential metabolites.

Supplementary Data 3: The significantly different metabolites between mOCs and mOCs^{Mito}. Untargeted metabolomics was performed between mOCs and mOCs^{Mito} (mOCs transplanted with osteolineage cell-derived mitochondria) and significantly different metabolites were annotated. The metabolites with VIP > 1 and P-value < 0.05 and fold change ≥2 or FC≤0.5 were considered to be differential metabolites.
